# Disease-driven reduction in human mobility influences human-mosquito contacts and dengue transmission dynamics

**DOI:** 10.1371/journal.pcbi.1008627

**Published:** 2021-01-19

**Authors:** Kathryn L. Schaber, T. Alex Perkins, Alun L. Lloyd, Lance A. Waller, Uriel Kitron, Valerie A. Paz-Soldan, John P. Elder, Alan L. Rothman, David J. Civitello, William H. Elson, Amy C. Morrison, Thomas W. Scott, Gonzalo M. Vazquez-Prokopec

**Affiliations:** 1 Program of Population Biology, Ecology and Evolution, Emory University, Atlanta, Georgia, United States of America; 2 Department of Biological Sciences and Eck Institute for Global Health, University of Notre Dame, Notre Dame, Indiana, United States of America; 3 Biomathematics Graduate Program and Department of Mathematics, North Carolina State University, Raleigh, North Carolina, United States of America; 4 Department of Biostatistics and Bioinformatics, Rollins School of Public Health, Emory University, Atlanta, Georgia, United States of America; 5 Department of Environmental Sciences, Emory University, Atlanta, Georgia, United States of America; 6 Department of Global Community Health and Behavioral Sciences, Tulane School of Public Health and Tropical Medicine, New Orleans, Louisiana, United States of America; 7 Graduate School of Public Health, San Diego State University, San Diego, California, United States of America; 8 Institute for Immunology and Informatics and Department of Cell and Molecular Biology, University of Rhode Island, Providence, Rhode Island, United States of America; 9 Department of Biology, Emory University, Atlanta, Georgia, United States of America; 10 Department of Entomology and Nematology, University of California Davis, Davis, California, United States of America; 11 Department of Pathology, Microbiology, and Immunology, School of Veterinary Medicine, University of California, Davis, California, United States of America; Institute for Disease Modeling, UNITED STATES

## Abstract

Heterogeneous exposure to mosquitoes determines an individual’s contribution to vector-borne pathogen transmission. Particularly for dengue virus (DENV), there is a major difficulty in quantifying human-vector contacts due to the unknown coupled effect of key heterogeneities. To test the hypothesis that the reduction of human out-of-home mobility due to dengue illness will significantly influence population-level dynamics and the structure of DENV transmission chains, we extended an existing modeling framework to include social structure, disease-driven mobility reductions, and heterogeneous transmissibility from different infectious groups. Compared to a baseline model, naïve to human pre-symptomatic infectiousness and disease-driven mobility changes, a model including both parameters predicted an increase of 37% in the probability of a DENV outbreak occurring; a model including mobility change alone predicted a 15.5% increase compared to the baseline model. At the individual level, models including mobility change led to a reduction of the importance of out-of-home onward transmission (*R*, the fraction of secondary cases predicted to be generated by an individual) by symptomatic individuals (up to -62%) at the expense of an increase in the relevance of their home (up to +40%). An individual’s positive contribution to *R* could be predicted by a GAM including a non-linear interaction between an individual’s biting suitability and the number of mosquitoes in their home (>10 mosquitoes and 0.6 individual attractiveness significantly increased *R*). We conclude that the complex fabric of social relationships and differential behavioral response to dengue illness cause the fraction of symptomatic DENV infections to concentrate transmission in specific locations, whereas asymptomatic carriers (including individuals in their pre-symptomatic period) move the virus throughout the landscape. Our findings point to the difficulty of focusing vector control interventions reactively on the home of symptomatic individuals, as this approach will fail to contain virus propagation by visitors to their house and asymptomatic carriers.

## Author summary

Human mobility patterns can play an integral role in vector-borne disease dynamics by characterizing an individual’s potential contacts with disease-transmitting vectors. Dengue virus is transmitted by a sedentary vector, but human mobility allows individuals to have contact with mosquitoes at their home and other houses they frequent (their activity space). When accounting for the decreased mobility of symptomatic dengue cases in an agent-based simulation model, however, we found a severely diminished role of the activity space in onward transmission. Those who received the majority of their mosquito contacts outside their home experienced decreases in expected bites and onward transmission when mobility changes were accounted for. Onward transmission was driven by a synergistic relationship between the number of mosquitoes in an individual’s home and their biting suitability, where even those with the highest biting suitability would have limited contribution to transmission given a low number of household mosquitoes. Reactive vector control, which often targets symptomatic cases, could be effective for slowing onward transmission from these cases, but will fail to control virus transmission due to the disproportionate contribution of asymptomatic infections.

## Introduction

The rate at which humans encounter vectors (mosquitoes, ticks, bugs) is a fundamental driver of vector-borne disease transmission dynamics [1,2]. Human-vector contacts can be influenced by a myriad of factors, including the vector’s host-seeking behavior [3,4], the host’s biting attractiveness to biting vectors [5–8], and the spatial distribution/density of both hosts and vectors [9–12]. Variations in some or all of these factors can lead to heterogeneous exposure, where certain individuals have higher contact rates with vectors than others [13–15]. The epidemiological consequence of such uneven distribution of human-vector contacts could be significant, particularly if it results in key encounters where a large number of vectors are infected [2]. Therefore, an individual’s contribution to transmission is influenced by not only how many vector bites are received, but also which vectors the bites are from and whom those vectors encounter next [16].

Given the central epidemiological role of mixing between hosts and vectors, there is a need for better quantification of its frequency and temporal variability, particularly because epidemiological outcomes depend on coupling among a variety of heterogeneities; i.e., human (behavior, immunity, etc.), vector (dispersal, longevity, etc.), and environmental [17]. Theoretical and simulation models have been used to assess the importance of such factors. One model focused on how heterogeneous exposure to vectors, poor mixing, and finite host numbers can determine the spatial scale of transmission [16]. Poor mixing can lead to infections being clustered in groups of closely connected individuals, as observed in the clustering of infections within social groups [18–20]. This association between human behavior and mixing is of particular relevance for dengue and other *Aedes*-borne viruses (dengue, chikungunya, Zika) [1,21–23], because house-to-house human movement, rather than mosquito mobility, is an underlying feature of spatial patterns of human incidence [18,20,24].

Dengue is an acute illness caused by any of four immunologically related viruses in the family *Flaviviridae* and transmitted by *Aedes* spp. mosquitoes (primarily *Aedes aegypti*). Prevalent in the tropics and subtropics, it is the most important mosquito-borne viral disease of humans worldwide [25]. Symptoms associated with dengue (acute fever, headache, musculoskeletal pain, and rash) only disrupt a person’s daily routine or result in treatment seeking in a small proportion of cases, whereas the other 70% of infected individuals experience either mild symptoms (inapparent) or no symptoms (asymptomatic) [26–28]. It has been recently shown that human mobility patterns change during the course of symptomatic (febrile) dengue infection. Specifically, symptomatic individuals visit fewer locations and stay at home more than when they were not infected [29–32]. Although disease-driven mobility changes have been shown to significantly influence the spread of directly transmitted pathogens, they have not yet been included in theoretical models of dengue virus (DENV) transmission [33,34]. For DENV, we hypothesize that the distribution of mosquitoes at an individual’s home and across the rest of the places they frequent (their activity space) will determine the impact of disease-driven mobility reductions on their mosquito contacts and onward transmission potential [17,21,32]. At a population level, we predict that human mobility changes could affect pathogen spread in a variety of ways depending upon which individuals in the population experience symptoms and change their mobility and potential exposure to *Aedes aegypti* mosquitoes.

For those DENV-infected individuals who experience symptoms, infectiousness tends to peak during the first few days after symptom onset when mobility is restricted and human-mosquito contacts are most likely occurring in the individual’s home [35–37]. There are, however, a few days before symptom onset when individuals have sufficient viremia levels to be infectious to mosquitoes, but have not yet changed their mobility [35,37]. A recent theoretical model of within-host viral dynamics estimated that 24% of an individual’s onward transmission results from mosquito bites during this pre-symptomatic phase [38]. We hypothesize that the pre-symptomatic period could have a significantly different contribution to onward transmission when accounting for mobility reductions, where individuals have normal mobility patterns during the pre-symptomatic period and decreased mobility during symptomatic infectiousness. To test our hypotheses, we examined the role of disease-driven mobility change in DENV transmission by theoretically exploring how day-to-day reductions in a symptomatic individual’s mobility and contacts with mosquitoes, combined with heterogeneous attractiveness to mosquitoes, may impact population-level DENV transmission dynamics.

## Methods

### Ethics statement

This manuscript is a theoretical modeling exercise and no field collected human or entomological data was included. The modeling was supported under human use protocol NAMRU-62014.0028 approved by the NAMRU-6 ethic committee and the Loreto Regional Health Department.

### Model framework

**Fig 1:**
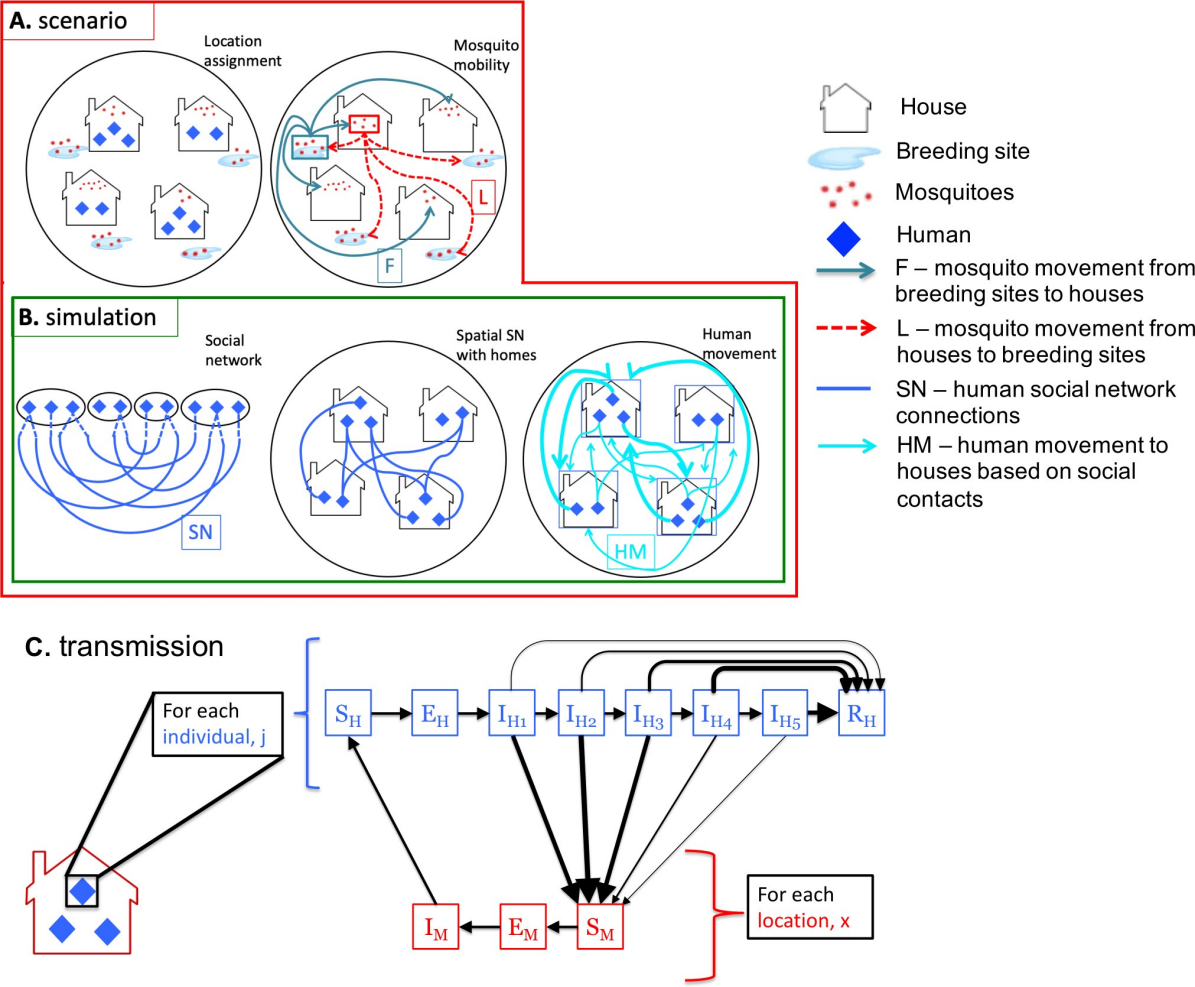
Diagram with setup of model framework for each scenario and simulation run. (A) For each scenario, this model includes houses and larval sites in which mosquitoes breed and move with specific daily probabilities. (B) For each of 200 simulation runs, a random social network (*SN*) is generated for humans, defining their contacts at home and at other houses and further determining the human movement matrix (*HM*). (C) Diagram of our stochastic compartmental transmission model in which mosquitoes at the household level were modeled as SEI (with M subscripts) and humans as an individual-based SEIR (with H subscripts). The I (infectious) stage for humans was divided into five sub-stages, each with their infectiousness value, shown here with weighted arrows. Individuals can either progress to the next (I_H*i*_) infectious sub-stage or move straight to the (R_H_) recovered stage based on a probability function. The probability of moving to the recovered stage is shown with weighted arrows (thicker arrows indicate higher weights).

Our model builds on a previously published mathematical framework that describes where and when human-mosquito contacts occur based on fine-scale human and mosquito mobility [16]. In the original framework, parameters with set values were defined (S1 Table), then a set of houses, {f}, and larval development sites, {l}, were arranged on a disc. Each house was assigned a number of residents equal to 2 plus a Poisson random variable (λ = 3.5), creating a two-person minimum per household. In order to assign the numbers of mosquitoes/larvae at each house/larval site, mosquito movement and reproduction were simulated for a total of 200 time steps, with the first 100 acting as a burn-in period. Counts of mosquitoes and larvae at each location were averaged over the second 100 time steps, providing the ‘equilibrium’ values (Fig 1A). Poorly mixed mosquito movement was characterized by matrices *L* and *F*, giving the distance-based probabilities of an adult female mosquito moving from any house to any larval site, and vice versa (Fig 1A and S2 Table) [16].

Rather than defining human mobility patterns based on distance (as in [16]), we generated a socially structured human mobility matrix for each of 200 simulation runs (Fig 1B). First, a random social network with household structure was constructed using the “configuration model” [39]. Each individual was assigned a number of “half-edges” (their degree) from a Poisson distribution with rate λ = 2.8, the mean number of residential locations visited in a data set described by Perkins et al. [32]. Based on human mobility data indicating that 14.7% of people do not regularly visit any houses (based on a 15 day monitoring period), a random sample of individuals predicted in the model to have a degree higher than 0 were reassigned a degree of 0 in order for the total modeled population to reach 15% of individuals with no half-edges and therefore no movement from their home [20]. Half-edges were then paired uniformly at random to form the edges of the social network, making sure there were no self-loops, multiple edges, or loops within houses (Fig 1B). This random network was represented as an |h|-by-|h| adjacency matrix *SN*, where |h| is the size of the set of hosts {h} (Table 1). A separate |h|-by-|f| presence/absence matrix, *Homes*, is constructed, where *Homes[j*,*x]* denotes whether or not the *j*^th^ host lives at the *x*^th^ residential site (Fig 1B and Table 1). Multiplying the *SN* and *Homes* matrices produced an |h|-by-|f| matrix, *HM*, denoting which residential sites an individual will visit based on their social network (note that this matrix was not presence/absence, as when the *j*^th^ host was socially connected to multiple individuals at the *x*^th^ residential site, *HM*[*j*,*x*] > 1) (Fig 1B and Table 1). This matrix was used to populate the human mobility matrix, *H*, which documented the proportion of time each host spent at each household (Table 1). Each host, *j*, spent 50% of their time at home (*H*[*j*, *home*] = 0.5) and divided the remaining 50% of their time into the houses visited in *HM* (When HM[*j*,*x*] > 1, as mentioned above, a proportionally larger amount of time was allocated at that residential location). For the 15% of individuals, *j*, who had no mobility outside their home *H*[*j*, *home*] = 1. As in the original model, each row of the *H* matrix described where a single host spent time and each column detailed all of the individuals spending time at a single household [16].

**Table 1:** Definitions of parameters mentioned in text.

Symbol	Type: Size	Definition
{f}	Vector: | f |	Number of houses
{l}	Vector: | l |	Number of larval sites
{h}	Vector: | h |	Number of hosts
c_i_	Vector: 5	Host-to-mosquito transmission efficiency for infectiousness stage I_i_
*L*	Matrix: | f | x | l |	Probability of mosquito movement from house to larval site
*F*	Matrix: | l | x | f |	Probability of mosquito movement from larval site to house
*SN*	Matrix: | h | x | h |	Random presence/absence social network
*Homes*	Matrix: | h | x | f |	Presence/absence matrix denoting where each host lives
*HM*	Matrix: | h | x | f |	Houses each host will visit based on *SN*
*H*	Matrix: | h | x | f |	Proportion of time each host spends at each house, based on *SN* and *HM*
*U*	Matrix: | f | x | h |	Distribution of mosquito bites on each host at each house
*B*_*norm*_	Matrix: | h | x | f |	Expected number of mosquito bites on each host at each house pre-epidemic
*B*_*i*_	Matrix: | h | x | f |	Expected number of mosquito bites on each host at each house for each infectiousness stage, i (I_1_ –I_5_)
*V*	Matrix: | h | x | h |	Expected secondary bites on each host arising from primary bites on all other hosts over one time step
*V*_*i*_	Matrix: | h | x | h |	Expected secondary bites on each host arising from primary bites on all other hosts at each time step of infectiousness, i (I_1_ –I_5_), accounting for mobility change
*R*_*norm*_	Matrix: | h | x | h |	Expected probability of a host receiving 1+ secondary infectious bites arising from primary infection of any other host
*R*_*movement*_	Matrix: | h | x | h |	Expected probability of a host receiving 1+ secondary infectious bites arising from primary infection of any other host, accounting for mobility changes
*R*_*x*_*(home)*	Matrix: | h | x | h |	Subset of R_x_ accounting for secondary cases that arise only from primary infectious bites at the primary host’s home (x = norm, movement)
*R*_*x*_*(other houses)*	Matrix: | h | x | h |	Subset of R_x_ accounting for secondary cases that arise from primary infectious bites that occur anywhere but at the primary host’s home (x = norm, movement)

Following the original model, each individual was also assigned a biting suitability score (which accounts for biting attractiveness, avoidance behavior, and defensive behavior) using a random exponential draw with rate based on empirical biting data [40]. Based on the mobility matrix, *H*, and biting suitability scores, an |f|-by-|h| matrix *U* was created to describe the distribution of mosquito bites on all individuals at each house, where each row gave the distribution of bites on all hosts at a single household and each column depicted the bites distributed on a single individual across all households (Table 1).

A stochastic transmission model was layered on top of this framework, which included a household-level SEI (susceptible, exposed, infectious) model for mosquitoes and an individual-based SEIR (susceptible, exposed, infectious, recovered) model for hosts (Fig 1C and S1 Fig). Individuals transitioned through one exposed (E) stage, based on pathogen latency of DENV in terms of feeding-cycle-length time steps. Hosts also transitioned through a maximum of five infectious (I_1_ –I_5_) sub-stages, until a stochastic transition into the recovered (R) stage. Rather than use a single set value for human infectiousness (as seen in Perkins et al.), values were chosen for each of these sub-stages (I_1_ –I_5_) based on data giving the mean daily probability of infection for mosquitoes after feeding on individuals with primary infections [16,38] (Fig 1C). For each 3-day infection time point in our model, we averaged these mean infectiousness values (Table 2). The updated transmission model also defined the first time step in the human infectiousness stage (I_1_) as the “pre-symptomatic period” and all subsequent infectious time steps (I_2_ –I_5_) as the “symptomatic” period, where the pre-symptomatic period contributed to 25% of infectiousness for individuals who progressed through all five infectiousness stage (I_1_ –I_5_) before recovery [38]. Simulation outbreaks were initiated by moving a single human into the first infectious (I) stage.

**Table 2:** Parameters that vary by infectiousness stage.

Stage of infectiousness	S	I_1_	I_2_	I_3_	I_4_	I_5_
**Day of Symptoms**	− − −	Pre-symptomatic	Days 1–3	Days 4–6	Days 7–9	Days 10–12
**Infectiousness**	− − −	0.4	0.7	0.4	0.1	0.01
**Time at home (%)**	50%	50%	100%	80%	70%	50%
**Fraction of original houses being visited**	1	1	0/3	1/3	2/3	1

Values calculated for individuals when susceptible, and at each sub-stage of infectiousness based on data from [31,38].

Model simulations had discrete time steps to capture the length of a mosquito gonotrophic cycle (~3 days). During each time step, hosts would allocate their time at houses based on *H*. The mosquitoes at each house would take blood meals from possible hosts (one blood meal per mosquito) based on *U* matrix probabilities and move to a larval site based on *L* probabilities. Eggs were laid based on a Poisson distribution with mean equal to number of adult females at the site multiplied by average egg batch size. Adult mosquitoes then moved to a house searching for their next blood meal based on *F* probabilities. During each time step, mosquito larvae also progressed through 4 developmental stages based on site-specific density dependence until emerging into adult mosquitoes. For both mosquitoes and humans, each time step also accounted for progression through incubation (E), infectiousness (I), and (for hosts) recovery (R). Virus transmission occurred from infectious hosts to susceptible mosquitoes and from infectious mosquitoes to susceptible hosts. At the end of each time point, host mobility was changed for those hosts who were symptomatically infectiousness.

Host Mobility Changes: Two different scenarios were considered to examine mobility changes: (1) no symptomatic movement change and (2) movement change throughout symptomatic infection. For scenario (1) no changes were made to the mobility matrix. For scenario (2), host mobility changes occurred at each 3-day time step of symptomatic infection based on recently published data on human mobility throughout symptomatic DENV infection [31] (Table 2 and S2 Fig). As data from Schaber et al. [31] were grouped as days 1–3, 4–6, and 7–9 after symptom onset, they corresponded to the I_2_, I_3_, and I_4_ stages here. When individuals were in the first three days after symptom onset, they were significantly more likely to spend *all* of their time at home and visit *no* other residential places. Accordingly, when an individual transitioned into symptomatic infection in the simulation (I_2_), their movement was completely stopped (*HM*[*j*,] = 0) and all time was spent at home (*HM*[*j*, *home*] = 1). During days 4–6 after symptom onset (sub-stage I_3_), individuals spent an average of 76% of time at home and visited approximately 1/3 of normally frequented places. On days 7–9 after symptom onset (sub-stage I_4_) time at home and fraction of places being visited averaged 69% and 2/3, respectively. Therefore, we set the time at home to be 80% (70%) for the I_3_ (I_4_) stage and had individuals visiting 1/3 (2/3) of their originally frequented houses (Table 2). The order in which houses were added back into an individual’s movements in stages I_3_ and I_4_ was determined by random sample where a house’s probability of being chosen was weighted by its original *HM* value. This made it more likely that individuals would resume visiting houses where they were socially connected to multiple residents. When individuals reached the I_5_ stage (days 10–12 after symptom onset), movement patterns and time at home were reset to original values (Table 2). At the end of each time step, once these movement changes were updated for all symptomatic individuals in the *HM* matrix, the *H* and *U* matrices were recalculated as described above.

The effect of the pre-symptomatic period was accounted for by including two more scenarios of interest: (1b) no movement changes and no pre-symptomatic period and (2b) movement change throughout symptomatic infection and no pre-symptomatic period. For these scenarios the first stage of infectiousness (I_1_), the pre-symptomatic period, was removed and individuals became symptomatic immediately after the incubation period (E), with infectiousness and movements corresponding to the I_2_ –I_5_ stages.

In order to simplify the model and the effects of mobility change, two simplifications were made: inapparent cases were left out and mobility was completely halted on the first three days of symptoms. Two further versions of scenario (2) were created to ascertain the robustness of the model to changes in these parameters. The impact of symptomatic mobility change in the presence of inapparent and asymptomatic infections was examined with scenario (2c), where only 30% of individuals (chosen from a random binomial draw) had symptomatic infection with mobility change. In scenario (2d), we assessed the sensitivity of model outputs to mobility changes the first three days of symptoms. Rather than having mobility completely stop after symptom onset, it was decreased to the same level as days 4–6, where 80% of time was spent at home and 1/3 of original houses were visited (S3 Table).

Model Outputs: Perkins et al. created multiple metrics to explore how mobile hosts and mosquitoes contribute to pathogen dispersal [16]. Of particular interest was the matrix *R*, which corresponded to the concept of effective reproductive number. This matrix gave the probability that a primary infection in one host will result in a secondary infection in some other host, where summing each row provided the number of expected secondary infections arising from a single individual. The *B* matrix was also utilized to measure the expected number of bites per time step on each host at each blood-feeding habitat (house). Each row of *B* provided the number of expected bites on a single individual at all households and each column gave the expected number of bites occurring on all individuals at a single household during one time step. At the population level, dynamics were examined using the simulation outputs of cumulative number of infections at each time step and number of infectious hosts at each time step. We utilized these original metrics and created versions that accounted for mobility change.

The *B* matrix could be used as a way to examine heterogeneity in human-mosquito contact rates, not only across hosts/locations, but also throughout an individual’s infectiousness period. This metric was derived to account for multiple sources of heterogeneous biting, namely spatial variation in biting intensity, hosts’ allocation of time at a location, and relative biting attractiveness of hosts at that location. Spatial variation in biting intensity was included as a numeric vector with the number of expected bites per feeding cycle at each house (based on average number of new adult females emerging at each larval site and their subsequent mobility to nearby houses). Multiplying this numeric vector by the distribution of bites across hosts at each site (*U*), which account for the latter two factors of heterogeneous biting, results in the *B* matrix. Because this metric was based on the *U* matrix, and therefore affected by the human mobility matrix (*H*), a list of *B* matrices was created to measure biting pre-epidemic (with normal movements) and during each time step of infectiousness. Within each simulation, *B*_*norm*_ was calculated for all individuals before infection spread began. During disease spread, *B*_*i*_[*j*,] was recorded for each host, *j*, at each infectiousness sub-stage (I_1_ –I_5_), *i*. This set of matrices gave us the expected number of mosquito bites on each host at each house throughout infectiousness/mobility changes (Table 1).

The previously-derived version of the *R* matrix, referred to as *R*_*norm*_, measured the probability of host *k* receiving one or more secondary infectious bites arising from primary infectious host, *j* (Table 1). This accounts for the primary infectious host transmitting the virus to a susceptible mosquito (the primary infectious bite) and that newly infectious mosquito then transmitting the virus to a susceptible host (the secondary infectious bite). The *R* metric was slightly adjusted to account for time-step-specific infectiousness where

$$R_{j,k}=1-e^{-bV(c_{1}+c_{2}+c_{3}+c_{4}+c_{5})}$$

with c_i_ values representing an individual’s time-step-specific infectiousness values. The *V* matrix gave the number of expected secondary bites on each host arising from primary bites on all other hosts over one time step, where each column provides the number of expected secondary bites received by a single individual from primary bites on all other hosts during one time step and each row described the number of expected secondary bites on all hosts from primary bites on a single individual. To derive the *V* matrix, we considered an individual, *k*, who received an expected number of (primary) bites per feeding cycle at each house based on the *B* matrix. The mosquitoes from this house then went on to make an expected number of (secondary) bites at all possible houses based on mosquito mobility matrices. These secondary bites were distributed among hosts according to the *U* matrix. Because the *U* matrix affected the *V* matrix, host mobility change was accounted for by creating a set of matrices, *V*_*i*_, for each sub-stage of infectiousness (I_1_ –I_5_) (Table 1). When host *j* was infectious in the simulation, their *V*_*i*_[*j*,] values were recorded for each I sub-stage (I_1_ –I_5_). At the end of the simulation run a matrix referred to as *R*_*movement*_ was created, where

$$R_{movement}=1-e^{-b(V_{1}c_{1}+V_{2}c_{2}+V_{3}c_{3}+V_{4}c_{4}+V_{5}c_{5})}.$$

In order to examine the importance of the location where the primary infectious bite occurs on host *j*, we also divided *R*_*movement*_ into two separate matrices, *R*_*movement*_ (*home*), and *R*_*movement*_ (*other houses*) (Table 1). This was done by calculating *V*_*i*_(*home*) and *V*_*i*_(*other houses*), which derived the number of expected secondary bites on each host arising from primary bites that occur at each time point of infectiousness, *i*, on all other hosts *at their home* and *everywhere but their home*, respectively. These *V*_*i*_ (*home*) and *V*_*i*_ (*other houses*) matrices were then used to derive *R*_*movement*_ (*home*) and *R*_*movement*_ (*other houses*), respectively. Similarly, *R*_*norm*_ was divided into *R*_*norm*_ (*home*) and *R*_*norm*_ (*other houses*) in order to compare the effect of where a primary infectious bite occurred when not accounting for mobility (Table 1).

A new metric that focused on the number of mosquitoes present in each individual’s home was also calculated. The number of mosquitoes in each individual’s home was recorded at the beginning of the simulation run (pre-epidemic) and at each time point of infectiousness for that individual. For each scenario a list was output with all of these metrics for each of 200 simulation runs.

### Data analysis

Analysis of simulation outputs had three main objectives: determining the effects of disease-driven mobility changes on (1) population-level outbreak dynamics (e.g., total infections, timing of infection peak, length of epidemic), (2) individual-level onward transmission (i.e., the number of secondary infections arising from a single individual), and (3) individual-level human-mosquito contacts (i.e., the number of mosquito bites on a single host at each location during each stage of infectiousness).

For the first objective, determining the effects of mobility change on population-level disease dynamics, we compared four scenarios: no mobility change and no pre-symptomatic period (baseline); no mobility change and pre-symptomatic period; mobility change and no pre-symptomatic period; mobility change and pre-symptomatic period. The effect of mobility changes could be determined by comparing the “no mobility change” and “mobility change” scenarios. To determine the role of the pre-symptomatic period when mobility changes occur, we compared the difference in ‘mobility change, no pre-symptomatic’ and ‘mobility change, pre-symptomatic’ to the difference in ‘no mobility change, no pre-symptomatic’ and ‘no mobility change, pre-symptomatic’ in order to account for the effect of removing one period of infectiousness (the pre-symptomatic period).

The number of infectious hosts at each time step was used to calculate the maximum infection prevalence, the time to maximum prevalence, and the length of the epidemic (when the number of infectious hosts was 0 without increasing again). The cumulative number of infections at each time step was utilized to record the total percent of the population infected in an epidemic, as well the time point when the percent of cumulative infections reached 10% and 65% (representing the time the epidemic starts to take off and starts to slow down). For the remaining two objectives, we focused our analyses on the scenario where a pre-symptomatic period was present and mobility changes were occurring. In order to determine the effect of these mobility changes on onward transmission, the *R*_*norm*_ and *R*_*movement*_ matrices were utilized. Row sums of *R*_*movement*_ and *R*_*norm*_ gave the expected number of secondary infectious bites arising from all primary bites on an individual host either with or without accounting for movement changes. Similarly, row sums of *R*_*movement*_(*home*), *R*_*movement*_(*other houses*), *R*_*norm*_(*home*), and *R*_*norm*_(*other houses*) determined the expected secondary bites arising from an individual due to only primary bites at their home or only primary bites at other houses (with and without movement changes). The distributions of *R*_*movement*_ and *R*_*norm*_ values were compared and both absolute (*R*_*abs_change*_) and relative change (*R*_*rel_change*_) were calculated to examine how accounting for mobility affects an individual’s *R*-value, where

$$R_{abs\_change}=R_{movement}-R_{norm}$$

and

$$R_{rel\_change}=\frac{R_{movement}-R_{norm}}{R_{norm}}.$$

Possible predictor variables for onward transmission were examined using generalized additive models (GAMs) [41]. Best-fit models were determined for *R*_*norm*_, *R*_*movement*_, *R*_*movement*_(*home*), *R*_*rel_change*_, and *R*_*rel_change*_(*home*). The variables considered as predictors were an individual’s biting suitability score (which accounts for biting attractiveness, avoidance behavior, and defensive behavior), the number of mosquitoes in their home, the number mosquitoes in their activity space pre-exposure (places they frequented), and the percent of expected mosquito bites that occur at their home pre-exposure (i.e., prior to being bitten by a virus-infected mosquito). Best-fit was determined with ΔAICc and the percent of deviance explained by each model.

For the third objective, we calculated the expected number of mosquito contacts for each individual pre-exposure and at each stage of infectiousness (I_1_ –I_5_). Expected counts were calculated as row sums of *B*_*norm*_ and each *B*_*i*_ matrix. For all individuals that experienced infection, the change in number of expected mosquito contacts was calculated for each infectiousness stage, as compared to pre-exposure. Percent change was also calculated to account for variation in healthy mosquito contact counts

$$\frac{B_{i}-B_{norm}}{B_{norm}}.$$

We examined the importance of these variations in healthy mosquito contacts for the entire population. Further, given the epidemiological significance of those with the top 20% of contacts [15], we also compared individuals with the top 20% of expected contacts pre-exposure to the rest of the population (bottom 80%). *B* matrices were also used to determine the percent of an individual’s mosquito contacts that occurred at their home. Generalized additive models (GAMs) were examined for change in expected mosquitoes contacts, both as a number and a percent. Predictors and methods for finding best-fit models are as mentioned above. All statistical analyses were performed in R 3.3.0 statistical computing software.

## Results

### Epidemic dynamics

**Fig 2:**
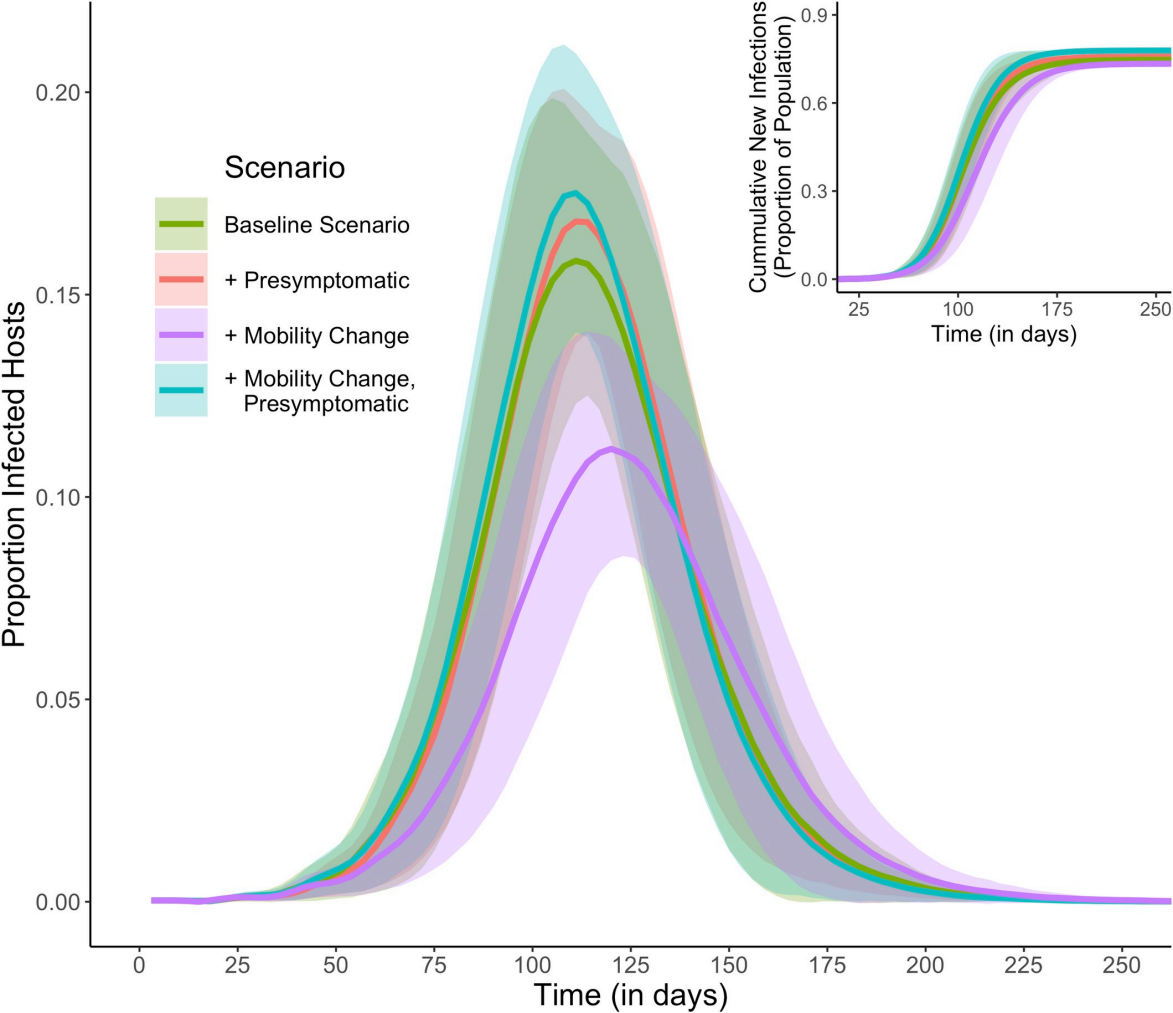
Predicted influence of disease-driven mobility reduction and pre-symptomatic transmission on DENV epidemic dynamics. For each scenario and for each time step, (main) the average proportion of infected hosts is calculated and (inset) the average proportion of cumulative infections is calculated. Averages are calculated across all simulation runs where an outbreak occurred, with standard deviations included in the shaded ribbons. The baseline scenario is one with no mobility change and no pre-symptomatic period.

Compared to a baseline model, naïve to pre-symptomatic infectiousness and disease-driven mobility change, a model including both parameters predicted an increase of 37% in the probability of a DENV outbreak occurring (from 39.5% to 76.0%) (Table 3). Models only including disease-driven mobility change or pre-symptomatic infectiousness increased the probability of an outbreak by 14% and 15.5%, respectively, compared to the baseline model (Table 3). In the simulations where outbreaks did not occur, the infection only spread to a few people (<1% of the population) before virus transmission ceased. For simulations leading to epidemics, the inclusion of pre-symptomatic infectiousness had minimal effects on parameters such as time to peak infection, the length of the epidemic, or how many individuals were infected (Table 3). Adding symptomatic mobility reductions to the baseline scenario had little effect on when the epidemic peaked or how long it lasted; however, there was a 5.2% decrease in peak transmission (Table 3). This reduction was compensated by accounting for infectiousness in the pre-symptomatic period (Fig 2).

**Table 3:** Dengue virus predicted infection prevalence based on presence of pre-symptomatic period and/or disease-driven mobility reductions, presented as mean ± 2SEM.

Scenarios*	Percent of Simulations Where Outbreak Occurred†	Maximum Percent Infection Prevalence	Days Until Maximum Prevalence	Days Until Epidemic End
Baseline Scenario	39.5%	19.0 ± 0.2%	113.0 ± 2.9	253.2 ± 6.0
+ Pre-symptomatic	53.5%	19.6 ± 0.1%	112.9 ± 2.1	259.1 ± 4.9
+ Mobility Changes	55.0%	13.8 ± 0.1%	122.9 ± 2.7	265.4 ± 4.9
+ Mobility Changes, Pre-symptomatic	76.0%	20.3 ± 0.1%	111.1 ± 1.8	250.3 ± 4.0

*Infection prevalence data were analyzed from four scenarios: (1) a baseline scenario with no mobility changes and no pre-symptomatic period, and alternate models that included (2) no mobility change and pre-symptomatic period included, (3) mobility change and no pre-symptomatic period, and (4) mobility change and pre-symptomatic period included. For each scenario, the average time point was listed for when infection prevalence reached its maximum and reached 0% at the end of epidemic. The percent of the population infected during maximum infection prevalence was also listed, as well as the number of simulations where an outbreak occurred. Time steps values were converted to days (1 time step = 3 days).

† Given as a percent out of 200 possible simulations.

### Onward transmission

**Fig 3:**
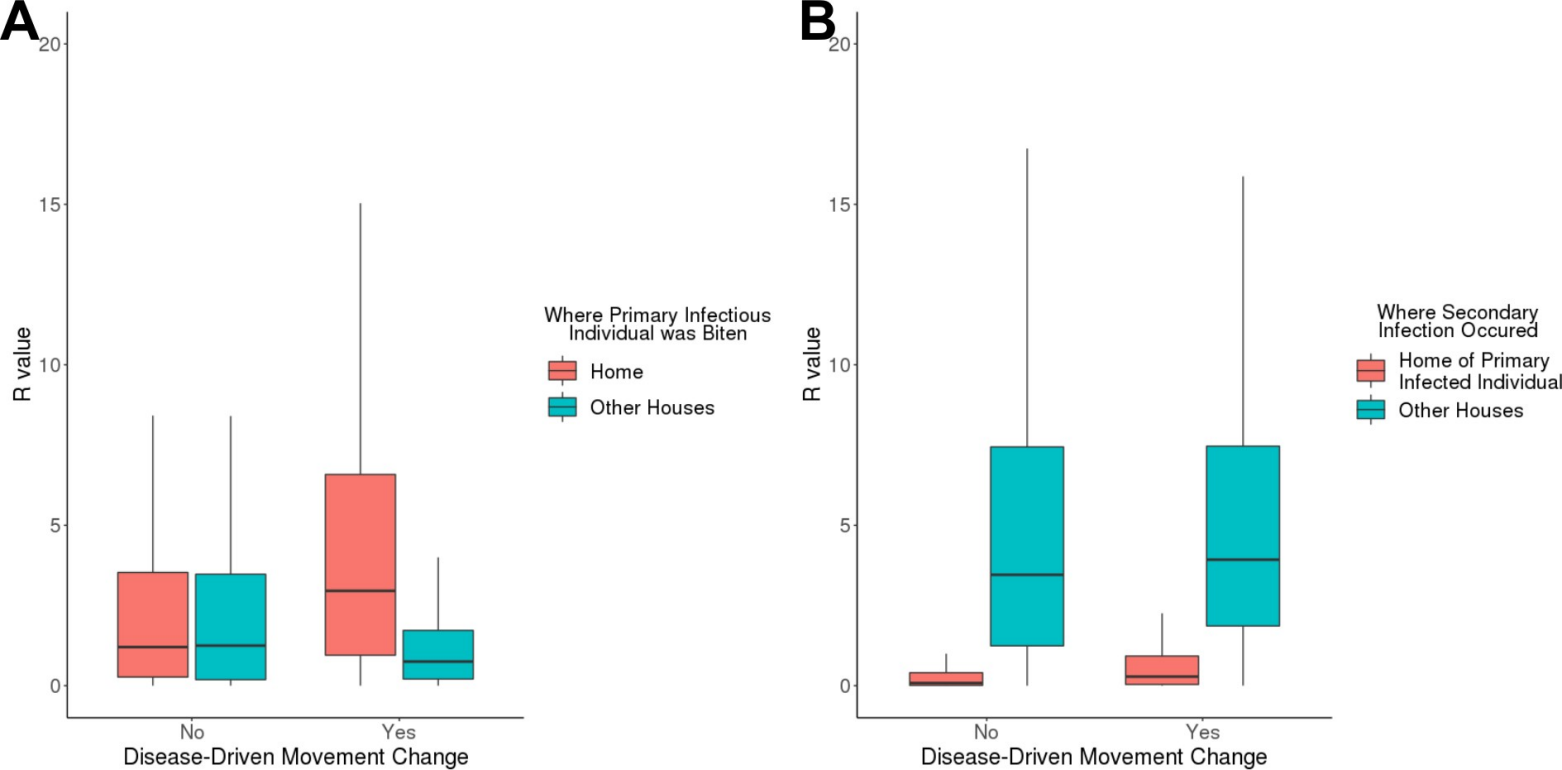
Expected onward transmission (*R*) values with and without mobility changes accounted for, separated by where primary bites occur and where secondary bites occur. (A) gives onward transmission for primary bites occurring at home (red) and at other houses (blue) both without (left) and with (right) movement change included. [from left to right: *R*_*norm*_(*home*), *R*_*norm*_(*other houses*), *R*_*movement*_(*home*), and *R*_*movement*_(*other houses*)] (B) gives onward transmission for secondary bites at the home of the primary infected individual (red) and at other houses (blue).

At the population level, the distributions of onward transmission (*R*) values increased slightly when disease-driven mobility reductions were added to a model without them (from an average ± SD of 5.4 ± 5.1 to 5.9 ± 4.8 infections) (Table 4 and S3 Fig). At the individual level, models including mobility change led to a reduction of the importance of out-of-home onward transmission by symptomatic individuals (reductions in *R* for primary and secondary bites of -62% and -17%, respectively) at the expense of an increase in the relevance of their home (increases in *R* for primary and secondary bites of 40% and 32%, respectively) (Table 4 and Fig 3). While the home environment played a key role in primary infections (Fig 3A), the majority of secondary infectious bites contributing to transmission occurred at other houses (Fig 3B).

**Table 4:** Average onward transmission (*R*) values with and without mobility change and change in *R* due to mobility change inclusion.

	Mean (sd) Onward Transmission		Mean (sd) Change in Individual Onward Transmission with Movement Changes	
	*R*_*norm*_	*R*_*movement*_	*R*_*abs_change*_	*R*_*rel_change*_
1° bites at home	2.3 (2.7)	4.3 (4.2)	1.0 (1.1)	39.74% (22.67)
1° bites at other houses	2.2 (2.6)	1.1 (1.2)	-1.9 (1.9)	-62.28% (9.13)
2° bites at infectious individual’s home	0.3 (0.4)	0.6 (0.7)	0.1 (0.2)	31.86% (35.59)
2° bites elsewhere	5.0 (4.7)	5.2 (4.2)	-1.5 (3.2)	-17.1% (28.97)
Total	5.4 (5.1)	5.9 (4.8)	-1.1 (2.5)	-15.14% (29.94)

*R*_*norm*_ values were calculated using an individual’s healthy movement patterns, while *R*_*movement*_ values accounted for changes in mobility throughout infectiousness. Changes in *R*-values due to mobility inclusion were calculated for each individual as a raw number (*R*_*abs_change*_) and as a percent of *R*_*norm*_ value (*R*_*rel_change*_). Overall *R*-values were listed, as well as *R*-values based on only primary bites occurring at home or at other houses.

**Fig 4:**
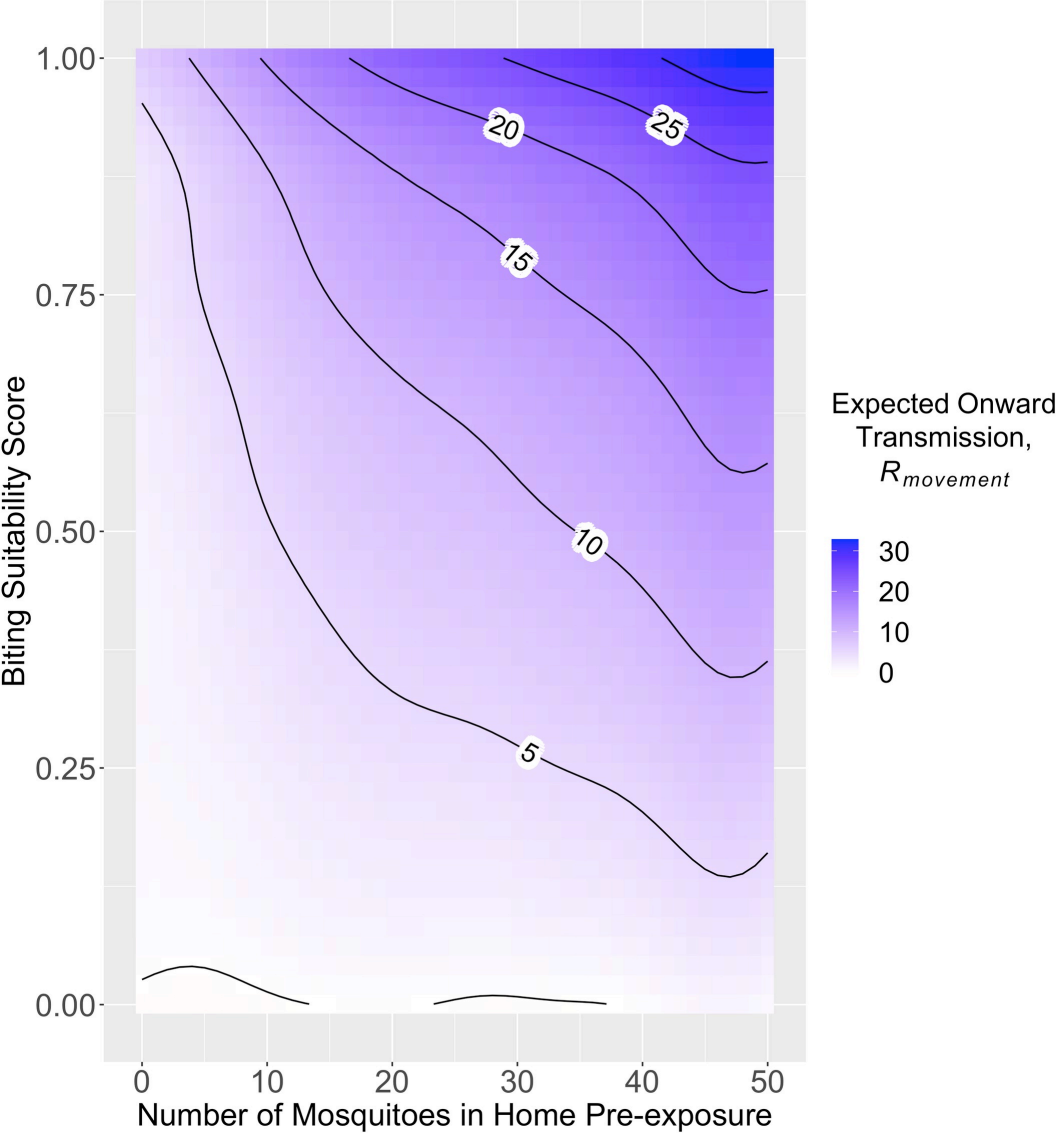
Predicted contribution to onward DENV transmission (*R*_*movement*_) based on a GAM with predictor variables number of mosquitoes in home pre-exposure, biting suitability score, and their interaction. The predicted values of onward transmission based on biting suitability and number of mosquitoes at home pre-exposure, presented as a heatmap with contours.

GAMs fitted to individual-level estimates of *R*_*movement*_ allowed understanding the mechanism by which mobility change influences onward transmission. In univariate GAMs, biting suitability score (which accounts for biting attractiveness, avoidance behavior, and defensive behavior) and number of mosquitoes at home pre-exposure explained 32.3% and 27.7% of deviance, respectively, whereas percent of bites expected at home pre-exposure only explained 9.3% of deviance (S5 Table). Increases in onward transmission were best explained by a GAM including an individual’s biting suitability and the number of mosquitoes in their home as well as their interaction (S5 Table and S4 Fig). Increasing biting suitability score from 0 to 1 only increased predicted *R*_*movement*_ by 5 new infections when there was a low mosquito count at home (e.g., 1–3 mosquitoes), compared to an increase of 30 new infections for those with high mosquito density (e.g., 40–50 mosquitoes) at home (Fig 4). In univariate GAMs for the two main parameters, including mobility change (*R*_*movement*_) increased dramatically the % deviance explained compared to models excluding it (*R*_*norm*_) (from 13.6% to 27.7% for number of mosquitoes at home and from 37.7% to 67.1% for biting suitability score) (S4 and S5 Tables). Including a variable for the total number of mosquitoes in all houses an individual visited pre-exposure (their activity space) did not increase the fit of GAMs (S4 Table).

**Fig 5:**
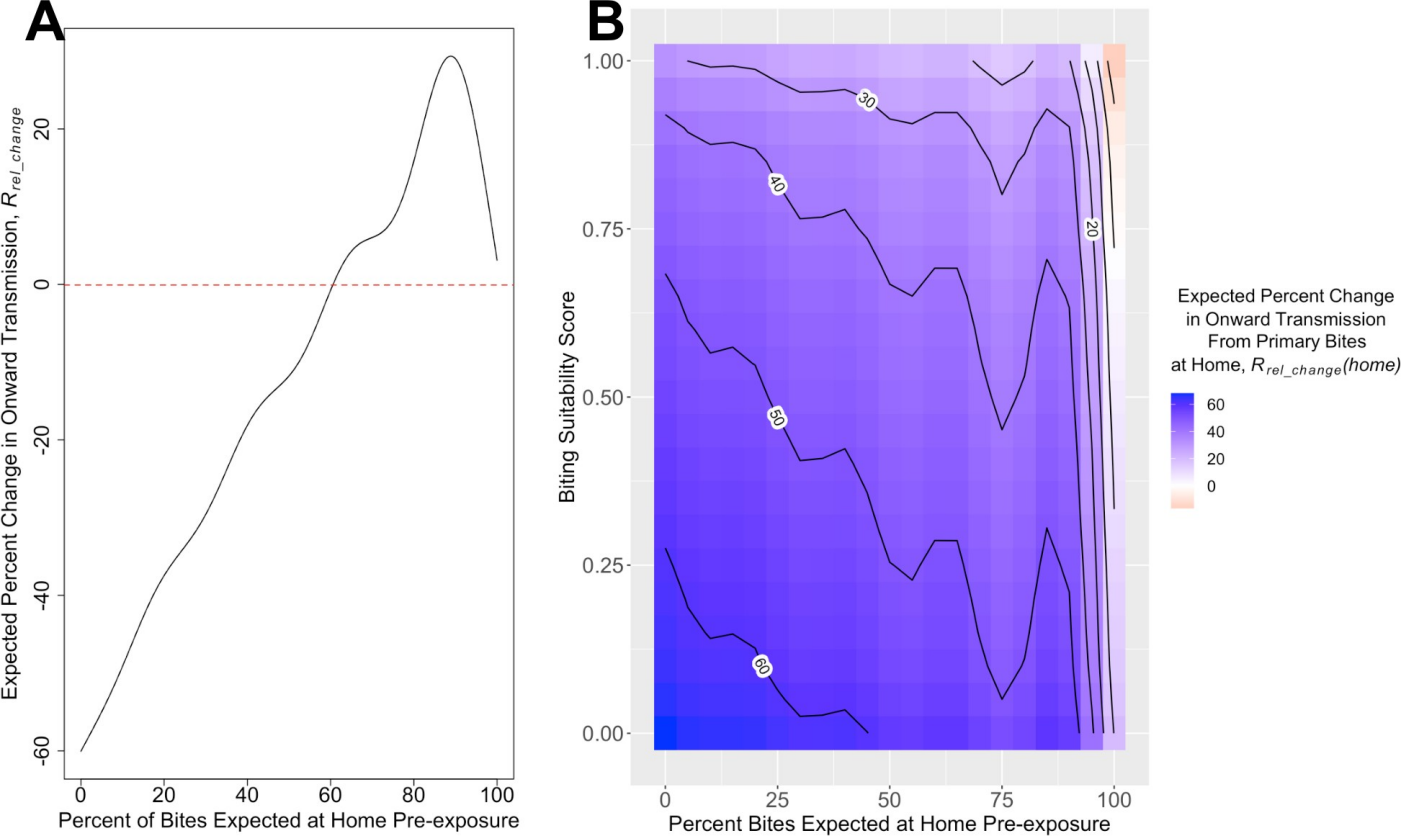
Predictions for percent change in expected onward transmission when mobility is included ((*R*_*rel_change*_ and *R*_*rel_change*_(*home*)) based on GAM models. (A)The predicted percent change in onward transmission (*R*_*rel_change*_) based on percent of bites expected at home pre-exposure. (B) The predicted percent change in onward transmission from primary bites at home *R*_*rel_change*_(*home*)) based on biting suitability and percent of bites expected at home pre-exposure, presented as a heatmap with contours.

Disease-driven mobility changes can either increase or decrease an individual’s contribution to onward transmission. GAMs were fitted to the percent change in onward transmission arising from all mosquito bites (*R*_*rel_change*_) and from only bites at home (*R*_*rel_change*_*(home)*). For both metrics, the best-fit GAM included biting suitability, percent of bites at home, their non-linear interaction, and the number of mosquitoes at home (S6 Table). For *R*_*rel_change*_ there was only a 3.0% loss in deviance explained when instead using a univariate GAM with percent of bites expected to occur at home pre-exposure (from 83.6% to 80.6%) (S6 Table). When the percent of bites expected to occur at home pre-exposure was below 61%, there was a predicted decrease in onward transmission, whereas those with greater than 61% of bites expected at home pre-exposure saw increases in onward transmission when mobility was accounted for (Fig 5A). The notable exception to this monotonically increasing effect was the tempered increase in onward transmission for those who received their pre-exposure bites almost exclusively at home (Fig 5A). When examining the percent change in onward transmission only from primary bites at home (*R*_*rel_change*_(*home*)), a majority of the deviance was explained in a model with percent bites expected at home pre-exposure and biting suitability score (S6 Table). The percent change in onward transmission from primary bites at home was predicted to be positive for all individuals, with the largest percent increase for individuals with low biting suitability scores and a small percent of bites expected to occur at home pre-exposure (Fig 5B).

### Human-mosquito contacts during illness

At the population level, the number of expected mosquito contacts was similarly distributed for each infectiousness sub-stage: before (I_1_), during (I_2_ –I_4_), and after (I_5_) mobility changes occurred (S7 Table and S5 Fig). When examining the change in an individual’s expected contacts at each infectiousness sub-stage during symptomatic mobility change, however, 57% of individuals had a decrease in expected contact and 38% had an increase (S6 and S7 Figs). During the first three days after symptom onset, the average change in expected contacts for individuals who received the top 20% and bottom 80% of expected mosquito contacts pre-exposure was a decrease of 13% and 17.5%, respectively (Table 5). Further, of those individuals who received the top 20% of expected mosquito contacts pre-exposure, 24% had a large enough decrease in mosquito contacts on the first three days after symptom onset to no longer be in the top 20% when symptomatic (S5 Fig).

**Table 5:** Average change in expected mosquito bites for each infectiousness sub-stage when symptomatic mobility changes are occurring (I_2_ –I_4_), separated based on expected bite values pre-exposure, provided as raw number and percent change relative to expected bites pre-exposure.

	Top 20% bites pre-exposure		Bottom 80% bites pre-exposure	
	Mean (sd) change in expected bites	Mean (sd) percent change in expected bites	Mean (sd) change in expected bites	Mean (sd) percent change in expected bites
Days 1–3 after symptom Onset	-0.9 (3.8)	-13.0% (48.9)	-0.2 (0.7)	-17.5% (51.0)
Days 4–6 after symptom Onset	-0.3 (2.7)	-5.6% (37.1)	-0.1 (0.5)	-8.7% (38.1)
Days 7–9 after symptom Onset	-0.1 (1.6)	-1.6% (22.1)	-0.1 (0.3)	-4.9% (23.2)

The percent change in expected mosquito contacts from pre-exposure to the first three days after symptom onset was best explained by a GAM including biting suitability score, percent of bites expected at home pre-exposure, number of mosquitoes at home pre-exposure, and the interaction between biting suitability and percent bites at home, which explained 93.4% of deviance (S8 Table). The model with only a term for percent of bites expected at home pre-exposure, however, was able to explain 92.1% of the deviance (S8 Table). The effect of percent bites at home on percent change in expected mosquito contacts was very similar to the effect on percent change in onward transmission, where those with less than 55% of bites at home pre-exposure had a predicted decrease in mosquito contacts during the first three days of illness and those with greater than 55% were predicted to see an increase in mosquito contacts (S8 Fig). Individuals who received none of their bites at home pre-exposure were expected to have the largest percent decrease, whereas those who received around 90% of their bites at home pre-exposure had the largest percent increase (S8 Fig). If change in expected mosquito contacts was examined as a raw value rather than a percent change, all three variables and the interaction between number of mosquitoes at home and percent of bites at home were needed to provide an accurate prediction and explain a large amount of the deviance (S8 Table).

### Sensitivity analyses

For the scenario where only 30% of cases experienced symptoms (and symptomatic mobility change), the expected values and relative changes for onward transmission and human-mosquito contacts had similar dynamics as in the case above where all individuals were symptomatic (S9–S15 Tables and S9–S13 Figs). In the scenario where symptomatic individuals did not completely halt mobility the first three days of symptoms, we saw similar albeit less intense dynamics as those above (S16–S22 Tables and S14–S18 Figs). This relaxing of mobility rules on the first days of symptoms did, however, curtail the importance of the household in onward transmission. When mobility was halted on days 1–3 there was a 40% increase in expected onward transmission from primary bites at home, as compared to a 30% increase with partial mobility on days 1–3 of symptoms (Tables 3 and S16 and S18 Fig). Similarly, the decrease in expected onward transmission from bites at other residential locations was 62% with no mobility on days 1–3 and 48% with partial mobility (Tables 3 and S16 and S18 Fig). Given this decreased role of the household in the case of partial mobility on the first three days of symptoms, the best-fit GAMs for predicting changes in onward transmission and changes in mosquito contacts could explain less deviance than their counterparts above (S17–S19 and S22 Tables). Regardless, we still saw the same predictor variables/reduced models with the largest contribution to explaining outcomes in the cases with partial and no mobility during days 1–3 after symptom onset (S17–S19 and S22 Tables).

## Discussion

Transmission of DENV is highly focal and dependent on key human*-Ae*. *aegypti* encounters [23,42–44]. Fine-scale human mobility expands the spatial scale of transmission and causes contacts to occur throughout an individual’s activity space (the houses they routinely visit), generating variation in exposure to mosquitoes [18,20,21,30,45]. Indeed, in the absence of mobility changes our models found primary bites at home and in the activity space both significantly contributed to an individual’s onward transmission potential. When more realism was incorporated into DENV simulation models, through the coupling between illness and human mobility and the addition of pre-symptomatic infectiousness, significant changes in the number of expected mosquito bites for an individual, the locations the bites occurred, the number of secondary cases they were expected to cause, and the overall epidemic dynamics were detected compared to models not including such parameters. Effects were apparent both at the population level, where the consideration of human pre-symptomatic infectiousness and disease-driven mobility change increased the probability of a DENV outbreak by 37%, and at the individual level where onward transmission increased in the home environment at the expense of the activity space, primarily driven by houses with high mosquito density and inhabited by individuals with a high biting attractiveness score. Our findings were robust to assumptions of the amount of human mobility change and the presence of asymptomatic infections, providing a mechanistic understanding of the effect of human movement in disease dynamics.

Symptom-driven reductions in mobility determined an individual’s onward transmission potential due to the increased role of mosquito contacts in the home and the diminished role of mosquitoes in the rest of the activity space. This shift in where mosquito contacts occurred while individuals were infectious led to both increased and decreased contact rates, largely based on what percent of mosquito contacts were already expected to occur at home before mobility changed. While the majority (57%) of individuals saw a decrease, those with greater than 55% of mosquito contacts in their home pre-exposure saw increases in expected mosquito contacts when mobility changes were present. These individuals were subsequently predicted to have relative increases in onward transmission. Those with a low percent of mosquito bites at home pre-exposure were predicted to see a decrease in overall onward transmission; however, they saw a relative increase in transmission from primary bites at home due to the increased time spent at home while infectious, as did those with low biting attractiveness scores. These changes in expected bites, and subsequently onward transmission, may be particularly important for superspreaders, those with the top 20% of expected mosquito contacts who are often targeted for control measures given their significant contributions to onward transmission. However, we found that a quarter (24%) of the individuals with the top 20% of expected contacts pre-exposure had drastic enough decreases in expected bites that they were no longer in the top 20% during infectiousness. Accordingly, a portion of individuals who weren’t identified before symptomatic mobility change entered the top 20% during the peak of their infectiousness (days 1–3 after symptom onset). Our models identify the problem in only focusing on individuals with high expected contact rates pre-exposure and suggest that if control targets are selected this way some potential superspreaders will likely be overlooked and control measures may be less efficacious than expected.

At the individual level, biting suitability has previously been recognized as an important determinant in onward transmission potential [1,2]. In our model, biting suitability accounts for host biting attractiveness as well as avoidance and defensive behaviors, leading to a distribution in the number of effective bites each individual is expected to receive. Our analysis further identified the significance of a synergistic interaction between biting suitability and the density of mosquitoes in an individual’s home. Those with only a few mosquitoes in their home (<10) could go from lowest to highest biting suitability score and cause only a couple more secondary infections, whereas those with large numbers of mosquitoes in their home (>30) could cause many more secondary cases. Furthermore, individuals with low values in either biting suitability score or number of mosquitoes at home were predicted to have low onward transmission, partially due to the interaction effect of these two variables. A recent study characterizing absolute indoor *Ae*. *Aegypti* abundance found an average of 12.9 (range: 1–169) females per house, indicating that the ranges of simulated densities and expected findings are within values characterized in endemic areas [46]. While biting suitability has been proposed as an important driver of DENV transmission dynamics [5,47], our study emphasizes that its role in onward DENV transmission prevails even in the presence of other sources of heterogeneity, such as disease-driven mobility change or human social structure.

While an individual’s biting suitability may not change, the number of mosquitoes in their home can, which has significant implications for disease control. Reducing household mosquitoes is predicted to decrease onward transmission for all individuals, but it would be particularly effective for those with high biting suitability given the synergistic effect of the two factors on expected transmission. In the case of reactive vector control, reducing mosquitoes at the home of a symptomatic individual would be important given that the majority of onward transmission is expected to stem from primary bites occurring in their home; however, only spraying the homes of symptomatic cases would fail to control virus transmission due to the prevalence of asymptomatic infections. In comparison, proactive vector control (where residual vector control is deployed prior to the onset of seasonal transmission and with high coverage) would lower an individual’s risk of becoming infected (due to decreased expected human-mosquito contacts in their home) and if infection did occur, both symptomatic and inapparent cases would be predicted to have decreased expected onward transmission due to the effect of vector control [48,49]. Moreover, reducing the number of mosquitoes in an individual’s home pre-infection could also decrease the percent of bites expected at home (relative to the rest of the activity space), meaning symptomatic mobility changes could further decrease expected onward transmission. In the presence of social distancing due to COVID-19, it is therefore critical to reduce household-level mosquito bites to prevent DENV onward transmission in the home environment as predicted by our model.

One limitation of our study was the lack of an empirical social network to accurately parameterize our model framework. However, the configuration model generates a random graph with a given degree sequence that exhibits the “small world” property [50], allowing us to account for the inhomogeneous nature of social interactions while also allowing conclusions to be generalized to multiple locations. Further, by wiring a new random social network at the beginning of each simulation, it’s unlikely that outcomes will be caused by specific artifacts of the network structure. The model was also limited by its size, being representative of a neighborhood rather than an entire city (due to computational limitations). However, given that the most significant effects were seen at the individual level (rather than the population-level), increasing the number of houses in the framework would likely not have a dramatic impact. Further research should focus on possible refinements of the model, including the impact of multiple dengue serotypes (which have different rates of infection in humans and infectiousness to mosquitoes), or the effect of heterogeneous mobility and biting attractiveness on vaccination and vector control strategies.

There are numerous factors that can contribute an individual’s onward transmission potential. In order to better understand the complex dynamics of DENV transmission, we developed a framework that examines the contribution of multiple heterogeneous factors, both individually and in relation to each other. In particular, the association between mobility and symptom severity was empirically parameterized to better understand its role in disease dynamics and to validate a conceptual model of the role of coupled heterogeneities in disease dynamics [17,51]. Symptomatic mobility change can have a significant impact on the relationship between biting suitability, density of mosquitoes, and location where the majority of mosquito contacts are occurring, leading to a spectrum of changes in expected mosquito contacts and onward transmission potential. It is necessary to account for the interconnectedness of these factors in order to understand the relative contribution of symptomatic individuals to overall epidemic transmission dynamics and predict the efficacy of control measures.

## Copyright statement

Author A.C.M. is a federal/contracted employee of the United States government. This work was prepared as part of her official duties. Title 17 U.S.C. §105 provides that ‘Copyright protection under this title is not available for any work of the United States Government.’ Title 17 U.S.C. §101 defines a U.S. Government work as a work prepared by a military service member or employee of the U.S. Government as part of that person’s official duties.

## Supporting information

S1 Table Simulation parameters with set values for all scenarios. (PDF)

S2 Table Simulation parameters set for each scenario. (PDF)

S3 Table Parameters that vary by infectiousness stage in the scenario with minor mobility the first three days after symptom onset. Values calculated for individuals when susceptible, and at each sub-stage of infectiousness based on data from [31,38].
(PDF)

S4 Table Comparison of GAMs for expected onward transmission without mobility change, *R*_*norm*_. Amount of deviance explained (%), degrees of freedom (DF), change in AICc compared to the best fit model (ΔAICc), and model weight are provided for each model. The best-fit model is highlighted in red.
(PDF)

S5 Table Comparison of GAMs for expected onward transmission. **Models are compared for response variables *R***_***movement***_ **and *R***_***movement***_**(*home*).** Amount of deviance explained (%), degrees of freedom (DF), change in AICc compared to the best fit model (ΔAICc), and model weight are provided for each model. The best-fit model is highlighted in red.
(PDF)

S6 Table Comparison of GAMs for percent change in expected onward transmission when mobility is included. **Models are compared for response variables *R***_***rel_change***_ **and *R***_***rel_change***_**(*home*).** Amount of deviance explained (%), degrees of freedom (DF), change in AICc compared to the best fit model (ΔAICc), and model weight are provided for each model. The best-fit model is highlighted in red.
(PDF)

S7 Table Average expected mosquito bites per person per time step for each sub-stage of infectiousness after symptom onset (I_2_ –I_5_), separated based on expected bite values pre-exposure, presented as mean (SD). (PDF)

S8 Table Comparison of GAMs for change in expected mosquito contacts in the first three days after symptom onset when all time is spent at home (sub-stage I_2_). Amount of deviance explained (%), degrees of freedom (DF), change in AICc compared to the best fit model (ΔAICc), and model weight are provided for each model. The best-fit model is highlighted in red.
(PDF)

S9 Table Average onward transmission (*R*) values with and without mobility change and change in *R* due to mobility change inclusion for only symptomatic individuals in the scenario with 70% inapparent cases and 30% symptomatic cases. *R*_*norm*_ values were calculated using an individual’s healthy movement patterns, while *R*_*movement*_ values accounted for changes in mobility throughout infectiousness. Changes in *R*-values due to mobility inclusion were calculated for each individual as a raw number and as a percent of *R*_*norm*_ value. Overall *R*-values were listed, as well as *R*-values based on only primary bites occurring at home or at other houses.
(PDF)

S10 Table Comparison of GAMs for expected onward transmission without mobility change, *R*_*norm*_, for only symptomatic individuals in the scenario with 70% inapparent cases and 30% symptomatic cases. Amount of deviance explained (%), degrees of freedom (DF), change in AICc compared to the best fit model (ΔAICc), and model weight are provided for each model. The best-fit model is highlighted in red.
(PDF)

S11 Table Comparison of GAMs for expected onward transmission for only symptomatic individuals in the scenario with 70% inapparent cases and 30% symptomatic cases. **Models are compared for response variables *R***_***movement***_ **and *R***_***movement***_**(*home*).** Amount of deviance explained (%), degrees of freedom (DF), change in AICc compared to the best fit model (ΔAICc), and model weight are provided for each model. The best-fit model is highlighted in red.
(PDF)

S12 Table Comparison of GAMs for percent change in expected onward transmission when mobility is included for only symptomatic individuals in the scenario with 70% inapparent cases and 30% symptomatic cases. **Models are compared for response variables *R***_***rel_change***_ **and *R***_***rel_change***_**(*home*).** Amount of deviance explained (%), degrees of freedom (DF), change in AICc compared to the best fit model (ΔAICc), and model weight are provided for each model. The best-fit model is highlighted in red.
(PDF)

S13 Table Average expected mosquito bites per person per time step for each sub-stage of infectiousness after symptom onset (I_2_ –I_5_) for only symptomatic individuals in the scenario with 70% inapparent cases and 30% symptomatic cases, separated based on expected bite values pre-exposure, presented as mean (SD). (PDF)

S14 Table Average change in expected mosquito bites for each infectiousness sub-stage when symptomatic mobility change is occurring (I_2_ –I_4_), for only symptomatic individuals in the scenario with 70% inapparent cases and 30% symptomatic cases, separated based on expected bite values pre-exposure. Average changes are given both as raw numbers and percent change relative to number of expected bites pre-exposure.
(PDF)

S15 Table Comparison of GAMs for change in expected mosquito contacts in the first three days after symptom onset when all time is spent at home (sub-stage I_2_) for only symptomatic individuals in the scenario with 70% inapparent cases and 30% symptomatic cases. **Models are compared for response variable as a raw number and a percentage.** Amount of deviance explained (%), degrees of freedom (DF), change in AICc compared to the best fit model (ΔAICc), and model weight are provided for each model. The best-fit model is highlighted in red.
(PDF)

S16 Table Average onward transmission (*R*) values with and without mobility change and change in *R* due to mobility change inclusion in the scenario with partial mobility on days 1–3 after symptom onset. *R*_*norm*_ values were calculated using an individual’s healthy movement patterns, while *R*_*movement*_ values accounted for changes in mobility throughout infectiousness. Changes in *R*-values due to mobility inclusion were calculated for each individual as a raw number and as a percent of *R*_*norm*_ value. Overall *R*-values were listed, as well as *R*-values based on only primary bites occurring at home or at other houses.
(PDF)

S17 Table Comparison of GAMs for expected onward transmission without mobility change, *R*_*norm*_, in the scenario with partial mobility on days 1–3 after symptom onset. Amount of deviance explained (%), degrees of freedom (DF), change in AICc compared to the best fit model (ΔAICc), and model weight are provided for each model. The best-fit model is highlighted in red.
(PDF)

S18 Table Comparison of GAMs for expected onward transmission in the scenario with partial mobility on days 1–3 after symptom onset. **Models are compared for response variables *R***_***movement***_ **and *R***_***movement***_**(*home*).** Amount of deviance explained (%), degrees of freedom (DF), change in AICc compared to the best fit model (ΔAICc), and model weight are provided for each model. The best-fit model is highlighted in red.
(PDF)

S19 Table Comparison of GAMs for percent change in expected onward transmission when mobility is included in the scenario with partial mobility on days 1–3 after symptom onset. **Models are compared for response variables *R***_***rel_change***_ **and *R***_***rel_change***_**(*home*).** Amount of deviance explained (%), degrees of freedom (DF), change in AICc compared to the best fit model (ΔAICc), and model weight are provided for each model. The best-fit model is highlighted in red.
(PDF)

S20 Table Average expected mosquito bites per person per time step for each sub-stage of infectiousness after symptom onset (I_2_ –I_5_) in the scenario with partial mobility on days 1–3 after symptom onset, separated based on expected bite values pre-exposure, presented as mean (SD). (PDF)

S21 Table Average change in expected mosquito bites for each infectiousness sub-stage when symptomatic mobility change is occurring (I_2_ –I_4_), in the scenario with partial mobility on days 1–3 after symptom onset, separated based on expected bite values pre-exposure. Average changes are given both as raw numbers and percent change relative to number of expected bites pre-exposure.
(PDF)

S22 Table Comparison of GAMs for change in expected mosquito contacts in the first three days after symptom onset when all time is spent at home (sub-stage I_2_) in the scenario with partial mobility on days 1–3 after symptom onset. **Models are compared for response variable as a raw number and a percentage.** Amount of deviance explained (%), degrees of freedom (DF), change in AICc compared to the best fit model (ΔAICc), and model weight are provided for each model. The best-fit model is highlighted in red.
(PDF)

S1 Fig Equations for human and mosquito transmission models, as seen in [16]. (A) Stochastic, individual-based SEIR dynamics for hosts. ρ_fail_ is a failure distribution that defines the probability of recovering after i time steps. (B) Stochastic, household-level SEI dynamics for mosquitoes. Bernoulli, Binomial, and Multinomial functions generate random numbers from those distributions with the supplied parameters. See **Tables 1 and** S1 **and** S2 for parameter definitions.
(PDF)

S2 Fig Mobility values during illness (in 3-day intervals), as seen in [31]. (A) Average number of locations visited per 3-day period. (B) Average proportion of time spent at home per 3-day period. Significant differences, denoted by letters, were detected using pairwise paired Wilcoxon Sign Rank tests with Bonferroni’s correction to account for a family-wise error-rate of 0.05. All significant differences had p-values < 0.05.
(PDF)

S3 Fig Distribution of *R*_*norm*_ and *R*_*movement*_ values. Outliers were removed.
(PDF)

S4 Fig Factors influencing the contribution of an individual to onward DENV transmission. Smooth functions for *R*_*movement*_ based on a GAM model containing number of mosquitoes in home pre-exposure, biting suitability score, and their interaction. The component smooths for each predictor variable are provided. For the 1-d smooths, the y-axis is the contribution of the predictor variable to the fitted response, centered around 0 (with 0 denoted by a red dashed line). For the 2-d smooth for the interaction term, a heatmap with overlaid contours is provided. The values of the contours represent the contribution of the interaction term to the fitted response. Positive values are in red and negative values are in blue.
(PDF)

S5 Fig Distribution of expected mosquito contacts at each infectiousness sub-stage, separated based on top 20/bottom 80% of expected bites pre-exposure. (PDF)

S6 Fig Distribution of change in expected mosquito contacts at each infectiousness sub-stage relative to pre-exposure values, separated based on top 20/bottom 80% of expected bites pre-exposure. (PDF)

S7 Fig Distribution of percent change in expected mosquito contacts at each infectiousness sub-stage relative to pre-exposure values, separated based on top 20/bottom 80% of expected bites pre-exposure. (PDF)

S8 Fig Predictions for percent change in expected mosquito contacts based on GAM model containing percent of bites expected at home pre-exposure. The predicted percent change in expected bites based on percent of bites expected at home pre-exposure. As there is only one predictor variable in this model, shifting the y-axis by the intercept value provides the smooth for the predictor variable.
(PDF)

S9 Fig Distribution of *R*_*norm*_ and *R*_*movement*_ values for only symptomatic individuals in the scenario with 70% inapparent cases and 30% symptomatic cases. Outliers were removed.
(PDF)

S10 Fig Expected onward transmission values with and without mobility changes accounted for, separated by where primary bites occur and where secondary bites occur, for only symptomatic individuals in the scenario with 70% inapparent cases and 30% symptomatic cases. (A) gives onward transmission for primary bites occurring at home (red) and at other houses (blue) both without (left) and with (right) movement change included. [from left to right: *R*_*norm*_(*home*), *R*_*norm*_(*other houses*), *R*_*movement*_(*home*), and *R*_*movement*_(*other houses*)] (B) gives onward transmission for secondary bites at the home of the primary infected individual (red) and at other houses (blue).
(PDF)

S11 Fig Distribution of expected mosquito contacts at each infectiousness sub-stage, for only symptomatic individuals in the scenario with 70% inapparent cases and 30% symptomatic cases, separated based on top 20/bottom 80% of expected bites pre-exposure. (PDF)

S12 Fig Distribution of change in expected mosquito contacts at each infectiousness sub-stage relative to pre-exposure values, for only symptomatic individuals in the scenario with 70% inapparent cases and 30% symptomatic cases, separated based on top 20/bottom 80% of expected bites pre-exposure. (PDF)

S13 Fig Distribution of percent change in expected mosquito contacts at each infectiousness sub-stage relative to pre-exposure values, for only symptomatic individuals in the scenario with 70% inapparent cases and 30% symptomatic cases, separated based on top 20/bottom 80% of expected bites pre-exposure. (PDF)

S14 Fig Distribution of *R*_*norm*_ and *R*_*movement*_ values in the scenario with partial mobility on days 1–3 after symptom onset. Outliers were removed.
(PDF)

S15 Fig Expected onward transmission values with and without mobility changes accounted for, separated by where primary bites occur and where secondary bites occur, in the scenario with partial mobility on days 1–3 after symptom onset. (A) gives onward transmission for primary bites occurring at home (red) and at other houses (blue) both without (left) and with (right) movement change included. [from left to right: *R*_*norm*_(*home*), *R*_*norm*_(*other houses*), *R*_*movement*_(*home*), and *R*_*movement*_(*other houses*)] (B) gives onward transmission for secondary bites at the home of the primary infected individual (red) and at other houses (blue).
(PDF)

S16 Fig Distribution of expected mosquito contacts at each infectiousness sub-stage in the scenario with partial mobility on days 1–3 after symptom onset, separated based on top 20/bottom 80% of expected bites pre-exposure. (PDF)

S17 Fig Distribution of change in expected mosquito contacts at each infectiousness sub-stage relative to pre-exposure values, in the scenario with partial mobility on days 1–3 after symptom onset, separated based on top 20/bottom 80% of expected bites pre-exposure. (PDF)

S18 Fig Distribution of percent change in expected mosquito contacts at each infectiousness sub-stage relative to pre-exposure values, in the scenario with partial mobility on days 1–3 after symptom onset, separated based on top 20/bottom 80% of expected bites pre-exposure. (PDF)

## References

[1] Dye C, Hasibeder G. Population dynamics of mosquito-borne disease: effects of flies which bite some people more frequently than others. Transactions of the Royal Society of Tropical Medicine and Hygiene. 1986;80(1):69–77. Epub 1986/01/01. 10.1016/0035-9203(86)90199-9 . 3727001

[2] Smith DL, Perkins TA, Reiner RC Jr., Barker CM, Niu T, Chaves LF, et al Recasting the theory of mosquito-borne pathogen transmission dynamics and control. Transactions of the Royal Society of Tropical Medicine and Hygiene. 2014;108(4):185–97. 10.1093/trstmh/tru026 24591453 PMC3952634

[3] Scott TW, Amerasinghe PH, Morrison AC, Lorenz LH, Clark GG, Strickman D, et al Longitudinal studies of Aedes aegypti (Diptera: Culicidae) in Thailand and Puerto Rico: blood feeding frequency. J Med Entomol. 2000;37(1):89–101. Epub 2004/06/29. 10.1603/0022-2585-37.1.89 . 15218911

[4] Chadee DD, Sutherland JM, Gilles JR. Diel sugar feeding and reproductive behaviours of Aedes aegypti mosquitoes in Trinidad: with implications for mass release of sterile mosquitoes. Acta Trop. 2014;132 Suppl:S86–90. 10.1016/j.actatropica.2013.09.019 . 24076041

[5] Liebman KA, Stoddard ST, Reiner RC Jr., Perkins TA, Astete H, Sihuincha M, et al Determinants of heterogeneous blood feeding patterns by Aedes aegypti in Iquitos, Peru. PLoS neglected tropical diseases. 2014;8(2):e2702 10.1371/journal.pntd.0002702 24551262 PMC3923725

[6] Verhulst NO, Andriessen R, Groenhagen U, Bukovinszkine Kiss G, Schulz S, Takken W, et al Differential attraction of malaria mosquitoes to volatile blends produced by human skin bacteria. PloS one. 2010;5(12):e15829 Epub 2011/01/07. 10.1371/journal.pone.0015829 21209854 PMC3012726

[7] Verhulst NO, Qiu YT, Beijleveld H, Maliepaard C, Knights D, Schulz S, et al Composition of human skin microbiota affects attractiveness to malaria mosquitoes. PloS one. 2011;6(12):e28991 Epub 2012/01/05. 10.1371/journal.pone.0028991 22216154 PMC3247224

[8] Port GR, Boreham PFL, Bryan JH. Relationship of host size to feeding mosquitoes of the Anopheles gambiae Giles complex (Diptera: Culicidae) [Vectors of disease in the Gambia]. 1980;v. 70.

[9] Bousema T, Griffin JT, Sauerwein RW, Smith DL, Churcher TS, Takken W, et al Hitting hotspots: spatial targeting of malaria for control and elimination. PLoS medicine. 2012;9(1):e1001165 10.1371/journal.pmed.1001165 22303287 PMC3269430

[10] Manore CA, Hickmann KS, Hyman JM, Foppa IM, Davis JK, Wesson DM, et al A network-patch methodology for adapting agent-based models for directly transmitted disease to mosquito-borne disease. J Biol Dyn. 2015;9:52–72. 10.1080/17513758.2015.1005698 25648061 PMC5473441

[11] Chao DL, Longini IM Jr., Halloran ME. The effects of vector movement and distribution in a mathematical model of dengue transmission. PloS one. 2013;8(10):e76044 10.1371/journal.pone.0076044 24204590 PMC3804532

[12] Padmanabha H, Correa F, Rubio C, Baeza A, Osorio S, Mendez J, et al Human Social Behavior and Demography Drive Patterns of Fine-Scale Dengue Transmission in Endemic Areas of Colombia. PloS one. 2015;10(12):e0144451 10.1371/journal.pone.0144451 26656072 PMC4684369

[13] Smith DL, McKenzie FE, Snow RW, Hay SI. Revisiting the basic reproductive number for malaria and its implications for malaria control. PLoS Biol. 2007;5(3):e42 Epub 2007/02/22. 10.1371/journal.pbio.0050042 17311470 PMC1802755

[14] Smith DL, Dushoff J, McKenzie FE. The risk of a mosquito-borne infection in a heterogeneous environment. PLoS Biol. 2004;2(11):e368 10.1371/journal.pbio.0020368 15510228 PMC524252

[15] Woolhouse MEJ, Dye C, Etard J-F, Smith T, Charlwood JD, Garnett GP, et al Heterogeneities in the transmission of infectious agents: Implications for the design of control programs. Proc Natl Acad Sci USA. 1997;94:338–42. 10.1073/pnas.94.1.338 8990210 PMC19338

[16] Perkins TA, Scott TW, Le Menach A, Smith DL. Heterogeneity, mixing, and the spatial scales of mosquito-borne pathogen transmission. PLoS computational biology. 2013;9(12):e1003327 10.1371/journal.pcbi.1003327 24348223 PMC3861021

[17] Vazquez-Prokopec GM, Perkins TA, Waller LA, Lloyd AL, Reiner RC Jr., Scott TW, et al Coupled Heterogeneities and Their Impact on Parasite Transmission and Control. Trends in parasitology. 2016;32(5):356–67. 10.1016/j.pt.2016.01.001 26850821 PMC4851872

[18] Reiner RC Jr., Stoddard ST, Scott TW. Socially structured human movement shapes dengue transmission despite the diffusive effect of mosquito dispersal. Epidemics. 2014;6:30–6. 10.1016/j.epidem.2013.12.003 24593919 PMC3971836

[19] Volz EM, Miller JC, Galvani A, Ancel Meyers L. Effects of heterogeneous and clustered contact patterns on infectious disease dynamics. PLoS computational biology. 2011;7(6):e1002042 Epub 2011/06/16. 10.1371/journal.pcbi.1002042 21673864 PMC3107246

[20] Stoddard ST, Forshey BM, Morrison AC, Paz Soldan V, Vazquez-Prokopec GM, Astete H, et al House-to-house human movement drives dengue virus transmission. Proc Natl Acad Sci. 2013;110(3):994–9. 10.1073/pnas.1213349110 23277539 PMC3549073

[21] Stoddard ST, Morrison AC, Vazquez-Prokopec GM, Paz Soldan V, Kochel TJ, Kitron U, et al The role of human movement in the transmission of vector-borne pathogens. PLoS neglected tropical diseases. 2009;3(7):e481 10.1371/journal.pntd.0000481 19621090 PMC2710008

[22] Wesolowski A, Qureshi T, Boni MF, Sundsoy PR, Johansson MA, Rasheed SB, et al Impact of human mobility on the emergence of dengue epidemics in Pakistan. Proceedings of the National Academy of Sciences of the United States of America. 2015;112(38):11887–92. 10.1073/pnas.1504964112 26351662 PMC4586847

[23] Salje H, Lessler J, Paul KK, Azman AS, Rahman MW, Rahman M, et al How social structures, space, and behaviors shape the spread of infectious diseases using chikungunya as a case study. Proceedings of the National Academy of Sciences of the United States of America. 2016;113(47):13420–5. 10.1073/pnas.1611391113 27821727 PMC5127331

[24] Vazquez-Prokopec GM, Bisanzio D, Stoddard ST, Paz-Soldan V, Morrison AC, Elder JP, et al Using GPS technology to quantify human mobility, dynamic contacts and infectious disease dynamics in a resource-poor urban environment. PloS one. 2013;8(4):e58802 10.1371/journal.pone.0058802 23577059 PMC3620113

[25] Bhatt S, Gething PW, Brady OJ, Messina JP, Farlow AW, Moyes CL, et al The global distribution and burden of dengue. Nature. 2013;496(7446):504–7. 10.1038/nature12060 23563266 PMC3651993

[26] WHO Guidelines Approved by the Guidelines Review Committee. Dengue: Guidelines for Diagnosis, Treatment, Prevention and Control: New Edition. Geneva: World Health Organization; World Health Organization; 2009.

[27] Kyle JL, Harris E. Global spread and persistence of dengue. Annual review of microbiology. 2008;62:71–92. 10.1146/annurev.micro.62.081307.163005 . 18429680

[28] Morrison AC, Minnick SL, Rocha C, Forshey BM, Stoddard ST, Getis A, et al Epidemiology of dengue virus in Iquitos, Peru 1999 to 2005: interepidemic and epidemic patterns of transmission. PLoS neglected tropical diseases. 2010;4(5):e670 10.1371/journal.pntd.0000670 20454609 PMC2864256

[29] Falcon-Lezama JA, Santos-Luna R, Roman-Perez S, Martinez-Vega RA, Herrera-Valdez MA, Kuri-Morales AF, et al Analysis of spatial mobility in subjects from a Dengue endemic urban locality in Morelos State, Mexico. PloS one. 2017;12(2):e0172313 10.1371/journal.pone.0172313 28225820 PMC5321279

[30] Arthur RF, Gurley ES, Salje H, Bloomfield LS, Jones JH. Contact structure, mobility, environmental impact and behaviour: the importance of social forces to infectious disease dynamics and disease ecology. Philos Trans R Soc Lond B Biol Sci. 2017;372(1719). 10.1098/rstb.2016.0454 28289265 PMC5352824

[31] Schaber KL, Paz-Soldan VA, Morrison AC, Elson WHD, Rothman AL, Mores CN, et al Dengue illness impacts daily human mobility patterns in Iquitos, Peru. PLoS neglected tropical diseases. 2019;13(9):e0007756 Epub 2019/09/24. 10.1371/journal.pntd.0007756 . 31545804 PMC6776364

[32] Perkins TA, Paz-Soldan VA, Stoddard ST, Morrison AC, Forshey BM, Long KC, et al Calling in sick: impacts of fever on intra-urban human mobility. Proc Biol Sci. 2016;283(1834). 10.1098/rspb.2016.0390 27412286 PMC4947886

[33] Poletto C, Tizzoni M, Colizza V. Human mobility and time spent at destination: impact on spatial epidemic spreading. J Theor Biol. 2013;338:41–58. 10.1016/j.jtbi.2013.08.032 . 24012488

[34] Meloni S, Perra N, Arenas A, Gomez S, Moreno Y, Vespignani A. Modeling human mobility responses to the large-scale spreading of infectious diseases. Scientific reports. 2011;1:62 10.1038/srep00062 22355581 PMC3216549

[35] Clapham HE, Tricou V, Van Vinh Chau N, Simmons CP, Ferguson NM. Within-host viral dynamics of dengue serotype 1 infection. Journal of the Royal Society, Interface / the Royal Society. 2014;11(96). 10.1098/rsif.2014.0094 24829280 PMC4032531

[36] Duong V, Lambrechts L, Paul RE, Ly S, Lay RS, Long KC, et al Asymptomatic humans transmit dengue virus to mosquitoes. Proceedings of the National Academy of Sciences of the United States of America. 2015;112(47):14688–93. Epub 2015/11/11. 10.1073/pnas.1508114112 26553981 PMC4664300

[37] Nguyet MN, Duong TH, Trung VT, Nguyen TH, Tran CN, Long VT, et al Host and viral features of human dengue cases shape the population of infected and infectious Aedes aegypti mosquitoes. Proceedings of the National Academy of Sciences of the United States of America. 2013;110(22):9072–7. Epub 2013/05/16. 10.1073/pnas.1303395110 23674683 PMC3670336

[38] Ten Bosch QA, Clapham HE, Lambrechts L, Duong V, Buchy P, Althouse BM, et al Contributions from the silent majority dominate dengue virus transmission. PLoS Pathog. 2018;14(5):e1006965 10.1371/journal.ppat.1006965 29723307 PMC5933708

[39] Molloy M, Reed B. A critical point for random graphs with a given degree sequence. Random Structures & Algorithms. 1995;6(2–3):161–80. 10.1002/rsa.3240060204

[40] De Benedictis J, Chow-Shaffer E, Costero A, Clark GG, Edman JD, Scott TW. Identification of the people from whom engorged Aedes aegypti took blood meals in Florida, Puerto Rico, using polymerase chain reaction-based DNA profiling. The American journal of tropical medicine and hygiene. 2003;68(4):437–46. Epub 2003/07/24. . 12875293

[41] Wood SN. Fast stable restricted maximum likelihood and marginal likelihood estimation of semiparametric generalized linear models. Journal of the Royal Statistical Society (B) 73(1):3–36.2011.

[42] Yoon IK, Getis A, Aldstadt J, Rothman AL, Tannitisupawong D, Koenraadt CJ, et al Fine scale spatiotemporal clustering of dengue virus transmission in children and Aedes aegypti in rural Thai villages. PLoS neglected tropical diseases. 2012;6(7):e1730 10.1371/journal.pntd.0001730 22816001 PMC3398976

[43] Mammen MP, Pimgate C, Koenraadt CJ, Rothman AL, Aldstadt J, Nisalak A, et al Spatial and temporal clustering of dengue virus transmission in Thai villages. PLoS medicine. 2008;5(11):e205 Epub 2008/11/07. 10.1371/journal.pmed.0050205 18986209 PMC2577695

[44] Anders KL, Nga le H, Thuy NT, Ngoc TV, Tam CT, Tai LT, et al Households as foci for dengue transmission in highly urban Vietnam. PLoS neglected tropical diseases. 2015;9(2):e0003528 10.1371/journal.pntd.0003528 25680106 PMC4332484

[45] Perchoux C, Chaix B, Cummins S, Kestens Y. Conceptualization and measurement of environmental exposure in epidemiology: accounting for activity space related to daily mobility. Health & place. 2013;21:86–93. 10.1016/j.healthplace.2013.01.005 . 23454664

[46] Koyoc-Cardeña E, Medina-Barreiro A, Cohuo-Rodríguez A, Pavía-Ruz N, Lenhart A, Ayora-Talavera G, et al Estimating absolute indoor density of Aedes aegypti using removal sampling. Parasit Vectors. 2019;12(1):250 Epub 2019/05/23. 10.1186/s13071-019-3503-y 31113454 PMC6528352

[47] Harrington LC, Fleisher A, Ruiz-Moreno D, Vermeylen F, Wa CV, Poulson RL, et al Heterogeneous feeding patterns of the dengue vector, Aedes aegypti, on individual human hosts in rural Thailand. PLoS neglected tropical diseases. 2014;8(8):e3048 10.1371/journal.pntd.0003048 25102306 PMC4125296

[48] Hladish TJ, Pearson CAB, Patricia Rojas D, Gomez-Dantes H, Halloran ME, Vazquez-Prokopec GM, et al Forecasting the effectiveness of indoor residual spraying for reducing dengue burden. PLoS neglected tropical diseases. 2018;12(6):e0006570 Epub 2018/06/26. 10.1371/journal.pntd.0006570 29939983 PMC6042783

[49] Cavany SM, España G, Lloyd AL, Waller LA, Kitron U, Astete H, et al Optimizing the deployment of ultra-low volume and targeted indoor residual spraying for dengue outbreak response. PLoS computational biology. 2020;16(4):e1007743 Epub 2020/04/21. 10.1371/journal.pcbi.1007743 32310958 PMC7200023

[50] de Sola Pool I, Kochen M. Contacts and influence. Social Networks. 1978;1(1):5–51. 10.1016/0378-8733(78)90011-4.

[51] Lloyd AL, Kitron U, Perkins TA, Vazquez-Prokopec GM, Waller LA. The basic reproductive number for disease systems with multiple coupled heterogeneities. Mathematical biosciences. 2020;321:108294 Epub 2019/12/15. 10.1016/j.mbs.2019.108294 . 31836567 PMC7905963

