# Supplementary material for: Disease-driven reduction in human mobility influences human-mosquito contacts and dengue transmission dynamics
Source: PLoS Comput Biol. 2021 Jan 19;17(1):e1008627. doi: 10.1371/journal.pcbi.1008627 (PMC7845972; doi:10.1371/journal.pcbi.1008627)
Supplement: S1 Table — (PDF) [file pcbi.1008627.s001.pdf]

Symbol	Value	Definition
T	200	Number of time steps in simulation
ν	10	Number of mosquito eggs per capita per feeding cycle
$\lvert f \rvert$	600	Number of houses
$\lvert l \rvert$	600	Number of aquatic habitats
ξ	4	Length of the larval stage (in feeding cycles)
s_L	0.9	Mosquito survival between blood feeding (houses) and egg laying
s_F	0.9	Mosquito survival between egg laying and blood feeding (houses)
σ	3	Pathogen incubation period in hosts
τ	1	Pathogen incubation period in mosquitoes
ρ_{max}	5	Maximum number of time steps for host infectiousness (I)
c_i		Host-to-mosquito transmission efficiency for infectiousness stage I_i
b	0.75	Mosquito-to-host transmission efficiency
