# Supplementary material for: Disease-driven reduction in human mobility influences human-mosquito contacts and dengue transmission dynamics
Source: PLoS Comput Biol. 2021 Jan 19;17(1):e1008627. doi: 10.1371/journal.pcbi.1008627 (PMC7845972; doi:10.1371/journal.pcbi.1008627)
Supplement: S2 Table — (PDF) [file pcbi.1008627.s002.pdf]

Symbol	Definition
α	Strength of density dependence on larval mosquitoes
Hh	Number of hosts per house
L	Probability of mosquito movement from house to larval site
F	Probability of mosquito movement from larval site to house
Smf	Number of susceptible adult mosquitoes at each house
Sml	Number of susceptible adult mosquitoes at each larval site
ρ_i	Probability of host recovery at each stage of infectiousness
γ	Host biting suitability
SN	Random presence/absence social network
HM	Houses each host will visit based on *SN*
H	Proportion of time each host spends at each household, based on *SN* and *HM*
