# Supplementary material for: Disease-driven reduction in human mobility influences human-mosquito contacts and dengue transmission dynamics
Source: PLoS Comput Biol. 2021 Jan 19;17(1):e1008627. doi: 10.1371/journal.pcbi.1008627 (PMC7845972; doi:10.1371/journal.pcbi.1008627)
Supplement: S3 Table — Values calculated for individuals when susceptible, and at each sub-stage of infectiousness based on data from [31,38]. (PDF) [file pcbi.1008627.s003.pdf]

**Stage of infectiousness**	**S**	**I_1**	**I_2**	**I_3**	**I_4**	**I_5**
**Day of Symptoms**	− − −	Pre-symptomatic	Days 1-3	Days 4-6	Days 7-9	Days 10-12
**Infectiousness**	− − −	0.4	0.7	0.4	0.1	0.01
**Time at home (%)**	50%	50%	80%	80%	70%	50%
**Fraction of original houses being visited**	1	1	1/3	1/3	2/3	1
