# Supplementary material for: Disease-driven reduction in human mobility influences human-mosquito contacts and dengue transmission dynamics
Source: PLoS Comput Biol. 2021 Jan 19;17(1):e1008627. doi: 10.1371/journal.pcbi.1008627 (PMC7845972; doi:10.1371/journal.pcbi.1008627)
Supplement: S4 Table — Amount of deviance explained (%), degrees of freedom (DF), change in AICc compared to the best fit model (ΔAICc), and model weight are provided for each model. The best-fit model is highlighted in red. (PDF) [file pcbi.1008627.s004.pdf]

	Total Onward Transmission Without Mobility Changes Included			
Factors	Deviance Explained (%)	df	Δ AICc	Weight
Percent bites at home	0.53%	10.915	4.33×10^5	<0.001
Number of mosquitoes at home	13.57%	10.949	2.60×10^5	<0.001
Number of mosquitoes in activity space	2.97%	10.999	3.81×10^5	<0.001
Biting suitability score	37.69%	10.973	4.23×10^5	<0.001
Biting suitability score, Number of mosquitoes at home, Number of mosquitoes in activity space, Percent bites at home	64.60%	37.925	5.06×10^4	<0.001
Biting suitability score, Number of mosquitoes at home, Number of mosquitoes in activity space, Percent bites at home, (Biting suitability score) X (Number of mosquitoes at home)	69.13%	52.782	0.0	1.0
Biting suitability score, Number of mosquitoes at home, Number of mosquitoes in activity space, Percent bites at home, (Biting suitability score) X (Percent bites at home)	65.40%	53.754	4.22×10^4	<0.001
Biting suitability score, Number of mosquitoes at home, Number of mosquitoes in activity space, Percent bites at home, (Number of mosquitoes at home) X (Percent bites at home)	67.25%	53.898	2.19×10^4	<0.001
