# Supplementary material for: Disease-driven reduction in human mobility influences human-mosquito contacts and dengue transmission dynamics
Source: PLoS Comput Biol. 2021 Jan 19;17(1):e1008627. doi: 10.1371/journal.pcbi.1008627 (PMC7845972; doi:10.1371/journal.pcbi.1008627)
Supplement: S5 Table — Models are compared for response variables Rmovement and Rmovement(home). Amount of deviance explained (%), degrees of freedom (DF), change in AICc compared to the best fit model (ΔAICc), and model weight are provided for each model. The best-fit model is highlighted in red. (PDF) [file pcbi.1008627.s005.pdf]

	Total Onward Transmission				Onward Transmission from 1° bites at home			
Factors	Deviance Explained (%)	df	Δ AICc	Weight	Deviance Explained (%)	df	Δ AICc	Weight
Percent bites at home	9.30%	10.965	4.74×10^5	<0.001	28.66%	10.997	4.94×10^5	<0.001
Number of mosquitoes at home	27.67%	10.958	3.90×10^5	<0.001	42.98%	10.974	4.11×10^5	<0.001
Biting suitability score	32.29%	10.952	3.65×10^5	<0.001	20.02%	10.873	5.36×10^5	<0.001
Biting suitability score, Number of mosquitoes at home, Percent bites at home	67.11%	28.819	9.78×10^4	<0.001	69.44%	28.872	1.80×10^5	<0.001
Biting suitability score, Number of mosquitoes at home, Percent bites at home, (Biting suitability score) X (Number of mosquitoes at home)	74.74%	43.614	0.0	1.0	81.19%	44.528	0.0	1.0
Biting suitability score, Number of mosquitoes at home, Percent bites at home, (Biting suitability score) X (Percent bites at home)	69.33%	44.530	7.19×10^4	<0.001	76.21%	44.737	8.70×10^4	<0.001
Biting suitability score, Number of mosquitoes at home, Percent bites at home, (Number of mosquitoes at home) X (Percent bites at home)	67.89%	44.924	8.90×10^4	<0.001	70.04%	44.207	1.73×10^5	<0.001
