# Supplementary material for: Disease-driven reduction in human mobility influences human-mosquito contacts and dengue transmission dynamics
Source: PLoS Comput Biol. 2021 Jan 19;17(1):e1008627. doi: 10.1371/journal.pcbi.1008627 (PMC7845972; doi:10.1371/journal.pcbi.1008627)
Supplement: S6 Table — Models are compared for response variables Rrel_change and Rrel_change(home). Amount of deviance explained (%), degrees of freedom (DF), change in AICc compared to the best fit model (ΔAICc), and model weight are provided for each model. The best-fit model is highlighted in red. (PDF) [file pcbi.1008627.s006.pdf]

	Percent Change in Total Onward Transmission				Percent Change in Onward Transmission from 1° bites at home			
Factors	Deviance Explained (%)	df	Δ AICc	Weight	Deviance Explained (%)	df	Δ AICc	Weight
Percent bites at home	80.55%	10.999	6.37×10^4	<0.001	40.24%	10.999	9.63×10^4	<0.001
Number of mosquitoes at home	46.67%	10.960	4.38×10^5	<0.001	4.78%	10.810	2.70×10^5	<0.001
Biting suitability score	2.49%	10.277	6.61×10^5	<0.001	11.65%	10.825	2.41×10^5	<0.001
Biting suitability score, Number of mosquitoes at home, Percent bites at home	82.08%	28.646	3.34×10^4	<0.001	52.63%	28.846	1.03×10^4	<0.001
Biting suitability score, Number of mosquitoes at home, Percent bites at home, (Biting suitability score) X (Number of mosquitoes at home)	82.91%	42.873	1.59×10^4	<0.001	52.83%	38.762	8.76×10^3	<0.001
Biting suitability score, Number of mosquitoes at home, Percent bites at home, (Biting suitability score) X (Percent bites at home)	83.63%	44.235	0.0	1.0	53.93%	44.231	0.0	1.0
Biting suitability score, Number of mosquitoes at home, Percent bites at home, (Number of mosquitoes at home) X (Percent bites at home)	82.22%	44.111	3.06×10^4	<0.001	52.79%	44.416	9.10×10^3	<0.001
