# Supplementary material for: Disease-driven reduction in human mobility influences human-mosquito contacts and dengue transmission dynamics
Source: PLoS Comput Biol. 2021 Jan 19;17(1):e1008627. doi: 10.1371/journal.pcbi.1008627 (PMC7845972; doi:10.1371/journal.pcbi.1008627)
Supplement: S7 Table — (PDF) [file pcbi.1008627.s007.pdf]

| | Top 20% bites pre-exposure | Bottom 80% bites pre-exposure |
|---|---|---|
| Pre-exposure | 7.6 (3.2) | 1.6 (1.1) |
| Days 1-3 after symptom Onset | 6.1 (4.4) | 1.2 (1.1) |
| Days 4-6 after symptom Onset | 6.7 (4.0) | 1.3 (1.1) |
| Days 7-9 after symptom Onset | 7.2 (3.5) | 1.5 (1.1) |
| Days 10-12 after symptom Onset | 7.6 (3.3) | 1.6 (1.1) |
