# Supplementary material for: Disease-driven reduction in human mobility influences human-mosquito contacts and dengue transmission dynamics
Source: PLoS Comput Biol. 2021 Jan 19;17(1):e1008627. doi: 10.1371/journal.pcbi.1008627 (PMC7845972; doi:10.1371/journal.pcbi.1008627)
Supplement: S8 Table — Amount of deviance explained (%), degrees of freedom (DF), change in AICc compared to the best fit model (ΔAICc), and model weight are provided for each model. The best-fit model is highlighted in red. (PDF) [file pcbi.1008627.s008.pdf]

	Change in Expected Mosquito Contacts				Percent Change in Expected Mosquito Contacts			
Factors	Deviance Explained (%)	df	Δ AICc	Weight	Deviance Explained (%)	df	Δ AICc	Weight
Percent bites at home	24.58%	10.998	1.28×10^5	<0.001	92.08%	11.000	6.63×10^4	<0.001
Number of mosquitoes at home	8.53%	10.053	1.99×10^5	<0.001	53.82%	10.958	7.17×10^5	<0.001
Biting suitability score	7.20%	10.956	2.04×10^5	<0.001	1.66%	10.141	9.95×10^5	<0.001
Biting suitability score, Number of mosquitoes at home, Percent bites at home	37.17%	28.962	6.06×10^4	<0.001	92.97%	28.646	2.23×10^4	<0.001
Biting suitability score, Number of mosquitoes at home, Percent bites at home, (Biting suitability score) X (Number of mosquitoes at home)	39.72%	42.620	4.53×10^4	<0.001	93.16%	43.992	1.20×10^4	<0.001
Biting suitability score, Number of mosquitoes at home, Percent bites at home, (Biting suitability score) X (Percent bites at home)	46.37%	44.832	2.18×10^3	<0.001	93.38%	44.317	0.0	1.0
Biting suitability score, Number of mosquitoes at home, Percent bites at home, (Number of mosquitoes at home) X (Percent bites at home)	46.69%	44.778	0.0	1.0	93.02%	43.086	1.98×10^4	<0.001
