# Supplementary material for: Disease-driven reduction in human mobility influences human-mosquito contacts and dengue transmission dynamics
Source: PLoS Comput Biol. 2021 Jan 19;17(1):e1008627. doi: 10.1371/journal.pcbi.1008627 (PMC7845972; doi:10.1371/journal.pcbi.1008627)
Supplement: S9 Table — Rnorm values were calculated using an individual’s healthy movement patterns, while Rmovement values accounted for changes in mobility throughout infectiousness. Changes in R-values due to mobility inclusion were calculated for each individual as a raw number and as a percent of Rnorm value. Overall R-values were listed, as well as R-values based on only primary bites occurring at home or at other houses. (PDF) [file pcbi.1008627.s009.pdf]

	Mean (sd) Onward Transmission		Mean (sd) Change in Onward Transmission with Movement Changes	
	R_{norm}	$R_{movement}$	R_{abs_change}	R_{rel_change}
1° bites at home	3.3 (3.3)	4.4 (4.2)	1.0 (1.1)	38.8 (21.0)
1° bites at other houses	3.1 (3.1)	1.2 (1.2)	-1.9 (1.9)	-62.3 (8.8)
2° bites at infectious individual's home	0.3 (0.4)	0.5 (0.6)	0.0 (0.1)	31.86 (35.59)
2° bites elsewhere	4.6 (4.3)	5.5 (4.3)	-0.6 (1.8)	-17.1 (28.97)
Total	7.2 (5.5)	6.0 (4.8)	-1.1 (2.4)	-15.3 (29.2)
