# Supplementary material for: Disease-driven reduction in human mobility influences human-mosquito contacts and dengue transmission dynamics
Source: PLoS Comput Biol. 2021 Jan 19;17(1):e1008627. doi: 10.1371/journal.pcbi.1008627 (PMC7845972; doi:10.1371/journal.pcbi.1008627)
Supplement: S10 Table — Amount of deviance explained (%), degrees of freedom (DF), change in AICc compared to the best fit model (ΔAICc), and model weight are provided for each model. The best-fit model is highlighted in red. (PDF) [file pcbi.1008627.s010.pdf]

| | Total Onward Transmission Without Mobility Changes Included | | | |
|---|---|---|---|---|
| Factors | Deviance Explained (%) | df | Δ AICc | Weight |
| Percent bites at home | 1.11% | 10.849 | $4.10 \times 10^5$ | <0.001 |
| Number of mosquitoes at home | 14.12% | 10.976 | $3.65 \times 10^5$ | <0.001 |
| Number of mosquitoes in activity space | 2.78% | 10.694 | $4.05 \times 10^5$ | <0.001 |
| Biting suitability score | 41.31% | 10.969 | $2.43 \times 10^5$ | <0.001 |
| Biting suitability score,<br>Number of mosquitoes at home,<br>Number of mosquitoes in activity space,<br>Percent bites at home | 67.43% | 37.909 | $5.45 \times 10^4$ | <0.001 |
| Biting suitability score,<br>Number of mosquitoes at home,<br>Number of mosquitoes in activity space,<br>Percent bites at home,<br>(Biting suitability score) X (Number of mosquitoes at home) | 72.53% | 52.877 | 0.0 | 1.0 |
| Biting suitability score,<br>Number of mosquitoes at home,<br>Number of mosquitoes in activity space,<br>Percent bites at home,<br>(Biting suitability score) X (Percent bites at home) | 68.56% | 53.765 | $4.32 \times 10^4$ | <0.001 |
| Biting suitability score,<br>Number of mosquitoes at home,<br>Number of mosquitoes in activity space,<br>Percent bites at home,<br>(Number of mosquitoes at home) X (Percent bites at home) | 69.26% | 53.764 | $3.60 \times 10^4$ | <0.001 |
