# Supplementary material for: Disease-driven reduction in human mobility influences human-mosquito contacts and dengue transmission dynamics
Source: PLoS Comput Biol. 2021 Jan 19;17(1):e1008627. doi: 10.1371/journal.pcbi.1008627 (PMC7845972; doi:10.1371/journal.pcbi.1008627)
Supplement: S11 Table — Models are compared for response variables Rmovement and Rmovement(home). Amount of deviance explained (%), degrees of freedom (DF), change in AICc compared to the best fit model (ΔAICc), and model weight are provided for each model. The best-fit model is highlighted in red. (PDF) [file pcbi.1008627.s011.pdf]

	Total Onward Transmission				Onward Transmission from 1° bites at home			
Factors	Deviance Explained (%)	df	Δ AICc	Weight	Deviance Explained (%)	df	Δ AICc	Weight
Percent bites at home	10.57%	10.863	1.35×10^5	<0.001	29.22%	10.924	1.38×10^5	<0.001
Number of mosquitoes at home	29.11%	10.784	1.13×10^5	<0.001	43.93%	10.841	1.15×10^5	<0.001
Biting suitability score	34.15%	10.823	1.05×10^5	<0.001	20.96%	10.156	1.48×10^5	<0.001
Biting suitability score, Number of mosquitoes at home, Percent bites at home	70.12%	28.580	2.99×10^4	<0.001	71.25%	28.480	5.15×10^4	<0.001
Biting suitability score, Number of mosquitoes at home, Percent bites at home, (Biting suitability score) X (Number of mosquitoes at home)	78.15%	41.359	0.0	1.0	83.20%	42.973	0.0	1.0
Biting suitability score, Number of mosquitoes at home, Percent bites at home, (Biting suitability score) X (Percent bites at home)	72.73%	43.812	2.12×10^4	<0.001	78.19%	44.119	2.50×10^4	<0.001
Biting suitability score, Number of mosquitoes at home, Percent bites at home, (Number of mosquitoes at home) X (Percent bites at home)	70.63%	44.508	2.83×10^4	<0.001	71.75%	41.929	4.97×10^4	<0.001
