# Supplementary material for: Disease-driven reduction in human mobility influences human-mosquito contacts and dengue transmission dynamics
Source: PLoS Comput Biol. 2021 Jan 19;17(1):e1008627. doi: 10.1371/journal.pcbi.1008627 (PMC7845972; doi:10.1371/journal.pcbi.1008627)
Supplement: S12 Table — Models are compared for response variables Rrel_change and Rrel_change(home). Amount of deviance explained (%), degrees of freedom (DF), change in AICc compared to the best fit model (ΔAICc), and model weight are provided for each model. The best-fit model is highlighted in red. (PDF) [file pcbi.1008627.s012.pdf]

	Percent Change in Total Onward Transmission				Percent Change in Onward Transmission from 1° bites at home			
Factors	Deviance Explained (%)	df	Δ AICc	Weight	Deviance Explained (%)	df	Δ AICc	Weight
Percent bites at home	82.38%	10.999	1.78×10^4	<0.001	44.05%	10.995	2.96×10^4	<0.001
Number of mosquitoes at home	49.45%	10.956	1.19×10^5	<0.001	4.84%	10.422	8.04×10^4	<0.001
Biting suitability score	2.50%	7.769	1.81×10^5	<0.001	12.48%	9.630	7.24×10^4	<0.001
Biting suitability score, Number of mosquitoes at home, Percent bites at home	83.83%	25.628	9.56×10^3	<0.001	57.44%	28.521	3.44×10^3	<0.001
Biting suitability score, Number of mosquitoes at home, Percent bites at home, (Biting suitability score) X (Number of mosquitoes at home)	84.60%	38.348	4.95×10^3	<0.001	57.58%	35.900	3.14×10^3	<0.001
Biting suitability score, Number of mosquitoes at home, Percent bites at home, (Biting suitability score) X (Percent bites at home)	85.38%	41.127	0.0	1.0	58.95%	41.963	0.0	1.0
Biting suitability score, Number of mosquitoes at home, Percent bites at home, (Number of mosquitoes at home) X (Percent bites at home)	84.04%	39.580	8.36×10^3	<0.001	57.57%	42.877	3.16×10^3	<0.001
