# Supplementary material for: Disease-driven reduction in human mobility influences human-mosquito contacts and dengue transmission dynamics
Source: PLoS Comput Biol. 2021 Jan 19;17(1):e1008627. doi: 10.1371/journal.pcbi.1008627 (PMC7845972; doi:10.1371/journal.pcbi.1008627)
Supplement: S13 Table — (PDF) [file pcbi.1008627.s013.pdf]

	Top 20% bites pre-exposure	Bottom 80% bites pre-exposure
Pre-exposure	7.4 (3.1)	1.6 (1.1)
Days 1-3 after symptom Onset	6.1 (4.2)	1.2 (1.1)
Days 4-6 after symptom Onset	6.6 (3.8)	1.4 (1.1)
Days 7-9 after symptom Onset	7.1 (3.3)	1.5 (1.1)
Days 10-12 after symptom Onset	7.5 (3.1)	1.6 (1.1)
