# Supplementary material for: Disease-driven reduction in human mobility influences human-mosquito contacts and dengue transmission dynamics
Source: PLoS Comput Biol. 2021 Jan 19;17(1):e1008627. doi: 10.1371/journal.pcbi.1008627 (PMC7845972; doi:10.1371/journal.pcbi.1008627)
Supplement: S14 Table — Average changes are given both as raw numbers and percent change relative to number of expected bites pre-exposure. (PDF) [file pcbi.1008627.s014.pdf]

	Top 20% bites pre-exposure		Bottom 80% bites pre-exposure	
	Mean (sd) change in expected bites	Mean (sd) percent change in expected bites	Mean (sd) change in expected bites	Mean (sd) percent change in expected bites
Days 1-3 after symptom Onset	-0.8 (3.6)	-12.6 (47.2)	-0.2 (0.7)	-17.3 (49.9)
Days 4-6 after symptom Onset	-0.3 (2.5)	-5.9 (35.5)	-0.1 (0.5)	-9.0 (36.9)
Days 7-9 after symptom Onset	-0.09 (1.5)	-2.0 (20.7)	-0.05 (0.3)	-5.0 (22.5)
