# Supplementary material for: Disease-driven reduction in human mobility influences human-mosquito contacts and dengue transmission dynamics
Source: PLoS Comput Biol. 2021 Jan 19;17(1):e1008627. doi: 10.1371/journal.pcbi.1008627 (PMC7845972; doi:10.1371/journal.pcbi.1008627)
Supplement: S15 Table — Models are compared for response variable as a raw number and a percentage. Amount of deviance explained (%), degrees of freedom (DF), change in AICc compared to the best fit model (ΔAICc), and model weight are provided for each model. The best-fit model is highlighted in red. (PDF) [file pcbi.1008627.s015.pdf]

	Change in Expected Mosquito Contacts				Percent Change in Expected Mosquito Contacts			
Factors	Deviance Explained (%)	df	Δ AICc	Weight	Deviance Explained (%)	df	Δ AICc	Weight
Percent bites at home	23.54%	10.962	3.48×10^4	<0.001	93.59%	11.000	2.02×10^4	<0.001
Number of mosquitoes at home	8.31%	10.402	5.21×10^4	<0.001	56.03%	10.951	2.04×10^5	<0.001
Biting suitability score	7.38%	10.886	5.30×10^4	<0.001	1.71%	8.392	2.80×10^5	<0.001
Biting suitability score, Number of mosquitoes at home, Percent bites at home	36.54%	28.872	1.71×10^4	<0.001	94.43%	25.907	6.86×10^3	<0.001
Biting suitability score, Number of mosquitoes at home, Percent bites at home, (Biting suitability score) X (Number of mosquitoes at home)	39.14%	40.747	1.31×10^4	<0.001	94.60%	39.624	4.01×10^3	<0.001
Biting suitability score, Number of mosquitoes at home, Percent bites at home, (Biting suitability score) X (Percent bites at home)	45.56%	44.163	2.49×10^3	<0.001	94.82%	41.467	0.0	1.0
Biting suitability score, Number of mosquitoes at home, Percent bites at home, (Number of mosquitoes at home) X (Percent bites at home)	46.96%	44.717	0.0	1.0	94.47%	39.000	6.25×10^3	<0.001
