# Supplementary material for: Disease-driven reduction in human mobility influences human-mosquito contacts and dengue transmission dynamics
Source: PLoS Comput Biol. 2021 Jan 19;17(1):e1008627. doi: 10.1371/journal.pcbi.1008627 (PMC7845972; doi:10.1371/journal.pcbi.1008627)
Supplement: S16 Table — Rnorm values were calculated using an individual’s healthy movement patterns, while Rmovement values accounted for changes in mobility throughout infectiousness. Changes in R-values due to mobility inclusion were calculated for each individual as a raw number and as a percent of Rnorm value. Overall R-values were listed, as well as R-values based on only primary bites occurring at home or at other houses. (PDF) [file pcbi.1008627.s016.pdf]

	Mean (sd) Onward Transmission		Mean (sd) Change in Onward Transmission with Movement Changes	
	R_{norm}	$R_{movement}$	R_{abs_change}	R_{rel_change}
1° bites at home	2.2 (2.4)	3.8 (3.6)	0.7 (0.9)	30.4 (19.7)
1° bites at other houses	2.1 (2.3)	1.4 (1.4)	-1.3 (1.5)	-48.4 (21.3)
2° bites at infectious individual's home	0.3 (0.4)	0.6 (0.6)	0.1 (0.2)	25.3 (30.2)
2° bites elsewhere	4.5 (4.2)	5.0 (3.9)	-1.0 (2.3)	-13.5 (25.2)
Total	5.0 (4.6)	5.7 (4.4)	-0.8 (1.9)	-11.2 (25.9)
