# Supplementary material for: Disease-driven reduction in human mobility influences human-mosquito contacts and dengue transmission dynamics
Source: PLoS Comput Biol. 2021 Jan 19;17(1):e1008627. doi: 10.1371/journal.pcbi.1008627 (PMC7845972; doi:10.1371/journal.pcbi.1008627)
Supplement: S17 Table — Amount of deviance explained (%), degrees of freedom (DF), change in AICc compared to the best fit model (ΔAICc), and model weight are provided for each model. The best-fit model is highlighted in red. (PDF) [file pcbi.1008627.s017.pdf]

	Total Onward Transmission Without Mobility Changes Included			
Factors	Deviance Explained (%)	df	Δ AICc	Weight
Percent bites at home	1.38%	10.966	4.37×10^5	<0.001
Number of mosquitoes at home	14.84%	10.000	3.86×10^5	<0.001
Number of mosquitoes in activity space	2.76%	10.986	4.33×10^5	<0.001
Biting suitability score	41.17%	10.979	2.56×10^5	<0.001
Biting suitability score, Number of mosquitoes at home, Number of mosquitoes in activity space, Percent bites at home	66.80%	36.961	5.61×10^4	<0.001
Biting suitability score, Number of mosquitoes at home, Number of mosquitoes in activity space, Percent bites at home, (Biting suitability score) X (Number of mosquitoes at home)	71.71%	52.299	0.0	1.0
Biting suitability score, Number of mosquitoes at home, Number of mosquitoes in activity space, Percent bites at home, (Biting suitability score) X (Percent bites at home)	67.93%	52.864	4.40×10^4	<0.001
Biting suitability score, Number of mosquitoes at home, Number of mosquitoes in activity space, Percent bites at home, (Number of mosquitoes at home) X (Percent bites at home)	68.12%	51.864	4.19×10^4	<0.001
