# Supplementary material for: Disease-driven reduction in human mobility influences human-mosquito contacts and dengue transmission dynamics
Source: PLoS Comput Biol. 2021 Jan 19;17(1):e1008627. doi: 10.1371/journal.pcbi.1008627 (PMC7845972; doi:10.1371/journal.pcbi.1008627)
Supplement: S18 Table — Models are compared for response variables Rmovement and Rmovement(home). Amount of deviance explained (%), degrees of freedom (DF), change in AICc compared to the best fit model (ΔAICc), and model weight are provided for each model. The best-fit model is highlighted in red. (PDF) [file pcbi.1008627.s018.pdf]

	Total Onward Transmission				Onward Transmission from 1° bites at home			
Factors	Deviance Explained (%)	df	Δ AICc	Weight	Deviance Explained (%)	df	Δ AICc	Weight
Percent bites at home	7.77%	10.979	4.33×10^5	<0.001	28.42%	10.990	4.59×10^5	<0.001
Number of mosquitoes at home	25.59%	9.995	3.59×10^5	<0.001	43.45%	10.999	3.78×10^5	<0.001
Biting suitability score	34.44%	10.968	3.16×10^5	<0.001	19.36%	15.398	5.00×10^5	<0.001
Biting suitability score, Number of mosquitoes at home, Percent bites at home	66.73%	27.719	8.26×10^4	<0.001	69.83%	38.881	1.62×10^5	<0.001
Biting suitability score, Number of mosquitoes at home, Percent bites at home, (Biting suitability score) X (Number of mosquitoes at home)	73.85%	43.543	0.0	1.0	81.17%	44.806	0.0	1.0
Biting suitability score, Number of mosquitoes at home, Percent bites at home, (Biting suitability score) X (Percent bites at home)	68.79%	43.584	6.07×10^4	<0.001	76.14%	44.829	8.13×10^4	<0.001
Biting suitability score, Number of mosquitoes at home, Percent bites at home, (Number of mosquitoes at home) X (Percent bites at home)	67.17%	43.778	7.82×10^4	<0.001	70.28%	42.240	1.57×10^5	<0.001
