# Supplementary material for: Disease-driven reduction in human mobility influences human-mosquito contacts and dengue transmission dynamics
Source: PLoS Comput Biol. 2021 Jan 19;17(1):e1008627. doi: 10.1371/journal.pcbi.1008627 (PMC7845972; doi:10.1371/journal.pcbi.1008627)
Supplement: S19 Table — Models are compared for response variables Rrel_change and Rrel_change(home). Amount of deviance explained (%), degrees of freedom (DF), change in AICc compared to the best fit model (ΔAICc), and model weight are provided for each model. The best-fit model is highlighted in red. (PDF) [file pcbi.1008627.s019.pdf]

	Percent Change in Total Onward Transmission				Percent Change in Onward Transmission from 1° bites at home			
Factors	Deviance Explained (%)	df	Δ AICc	Weight	Deviance Explained (%)	df	Δ AICc	Weight
Percent bites at home	60.66%	10.998	2.16×10^4	<0.001	31.08%	10.998	5.40×10^4	<0.001
Number of mosquitoes at home	34.98%	10.973	1.94×10^5	<0.001	3.48%	10.778	1.70×10^5	<0.001
Biting suitability score	2.25%	9.434	3.34×10^5	<0.001	8.41%	10.692	1.52×10^5	<0.001
Biting suitability score, Number of mosquitoes at home, Percent bites at home	61.94%	27.354	1.02×10^4	<0.001	40.24%	28.810	5.00×10^3	<0.001
Biting suitability score, Number of mosquitoes at home, Percent bites at home, (Biting suitability score) X (Number of mosquitoes at home)	62.56%	40.085	4.60×10^3	<0.001	40.39%	39.313	4.14×10^3	<0.001
Biting suitability score, Number of mosquitoes at home, Percent bites at home, (Biting suitability score) X (Percent bites at home)	63.06%	42.407	0.0	1.0	41.11%	43.485	0.0	1.0
Biting suitability score, Number of mosquitoes at home, Percent bites at home, (Number of mosquitoes at home) X (Percent bites at home)	62.06%	32.323	9.19×10^3	<0.001	40.51%	42.627	3.50×10^3	<0.001
