# Supplementary material for: Disease-driven reduction in human mobility influences human-mosquito contacts and dengue transmission dynamics
Source: PLoS Comput Biol. 2021 Jan 19;17(1):e1008627. doi: 10.1371/journal.pcbi.1008627 (PMC7845972; doi:10.1371/journal.pcbi.1008627)
Supplement: S21 Table — Average changes are given both as raw numbers and percent change relative to number of expected bites pre-exposure. (PDF) [file pcbi.1008627.s021.pdf]

	Top 20% bites pre-exposure		Bottom 80% bites pre-exposure	
	Mean (sd) change in expected bites	Mean (sd) percent change in expected bites	Mean (sd) change in expected bites	Mean (sd) percent change in expected bites
Days 1-3 after symptom Onset	-0.2 (2.2)	-5.2 (35.6)	-0.1 (0.4)	-8.5 (36.6)
Days 4-6 after symptom Onset	-0.2 (2.3)	-5.1 (35.7)	-0.1 (0.4)	-8.4 (36.6)
Days 7-9 after symptom Onset	-0.04 (1.4)	-1.5 (21.4)	-0.05 (0.3)	-4.8 (22.2)
