# Supplementary material for: Disease-driven reduction in human mobility influences human-mosquito contacts and dengue transmission dynamics
Source: PLoS Comput Biol. 2021 Jan 19;17(1):e1008627. doi: 10.1371/journal.pcbi.1008627 (PMC7845972; doi:10.1371/journal.pcbi.1008627)
Supplement: S22 Table — Models are compared for response variable as a raw number and a percentage. Amount of deviance explained (%), degrees of freedom (DF), change in AICc compared to the best fit model (ΔAICc), and model weight are provided for each model. The best-fit model is highlighted in red. (PDF) [file pcbi.1008627.s022.pdf]

	Change in Expected Mosquito Contacts				Percent Change in Expected Mosquito Contacts			
Factors	Deviance Explained (%)	df	Δ AICc	Weight	Deviance Explained (%)	df	Δ AICc	Weight
Percent bites at home	25.65%	10.997	8.81×10^4	<0.001	68.69%	10.999	8.68×10^3	<0.001
Number of mosquitoes at home	12.47%	10.382	1.44×10^5	<0.001	40.05%	10.963	2.31×10^5	<0.001
Biting suitability score	5.25%	10.950	1.71×10^5	<0.001	1.31%	9.582	4.01×10^5	<0.001
Biting suitability score, Number of mosquitoes at home, Percent bites at home	33.66%	28.939	4.91×10^4	<0.001	69.24%	22.159	2.67×10^3	<0.001
Biting suitability score, Number of mosquitoes at home, Percent bites at home, (Biting suitability score) X (Number of mosquitoes at home)	37.34%	43.066	2.96×10^4	<0.001	69.35%	31.732	1.45×10^3	<0.001
Biting suitability score, Number of mosquitoes at home, Percent bites at home, (Biting suitability score) X (Percent bites at home)	42.54%	44.852	0.0	1.0	69.48%	36.984	0.0	1.0
Biting suitability score, Number of mosquitoes at home, Percent bites at home, (Number of mosquitoes at home) X (Percent bites at home)	38.81%	44.058	2.15×10^4	<0.001	69.34%	39.735	1.59×10^3	<0.001
