# Supplementary material for: Disease-driven reduction in human mobility influences human-mosquito contacts and dengue transmission dynamics
Source: PLoS Comput Biol. 2021 Jan 19;17(1):e1008627. doi: 10.1371/journal.pcbi.1008627 (PMC7845972; doi:10.1371/journal.pcbi.1008627)
Supplement: S1 Fig — (A) Stochastic, individual-based SEIR dynamics for hosts. ρfail is a failure distribution that defines the probability of recovering after i time steps. (B) Stochastic, household-level SEI dynamics for mosquitoes. Bernoulli, Binomial, and Multinomial functions generate random numbers from those distributions with the supplied parameters. See Tables 1 and S1 and S2 for parameter definitions. (PDF) [file pcbi.1008627.s023.pdf]

A

$$S(t+1)=\mathrm{Bernoulli}\left(S(t),(1-b)^{\sum_{\{f\}}\mathrm{Multinomial}(I'(t),U)}\right)$$

$$E_0(t+1)=S(t)-S(t+1)$$

$$E_e(t+1)=E_{e-1}(t)$$

$$I_0(t+1)=E_{\sigma-1}(t)$$

$$I_i(t+1)=\mathrm{Bernoulli}(I_{i-1}(t),1-\rho_{\mathrm{fail}}(i-1))$$

$$R(t+1)=R(t)+\left(\sum I_i(t)+E_{\sigma-1}(t)-\sum I_i(t+1)\right)$$

B

$$S'(t+1)=\mathrm{Multinomial}(A_\xi(t+1),F)+\mathrm{Multinomial}(S''(t),LF)$$

$$S''(t+1)=S'(t+1)-\mathrm{Binomial}(\mathrm{Multinomial}(S'(t+1),U),cI(t))$$

$$E'_0(t+1)=S'(t+1)-S''(t+1)$$

$$E'_e(t+1)=\mathrm{Multinomial}(E'_{e-1}(t),LF)$$

$$I'(t+1)=\mathrm{Multinomial}(I'(t)+E'_{\tau-1}(t),LF)$$
