# Supplementary figures and images for: Disease-driven reduction in human mobility influences human-mosquito contacts and dengue transmission dynamics
Source: PLoS Comput Biol. 2021 Jan 19;17(1):e1008627. doi: 10.1371/journal.pcbi.1008627 (PMC7845972; doi:10.1371/journal.pcbi.1008627)

**A**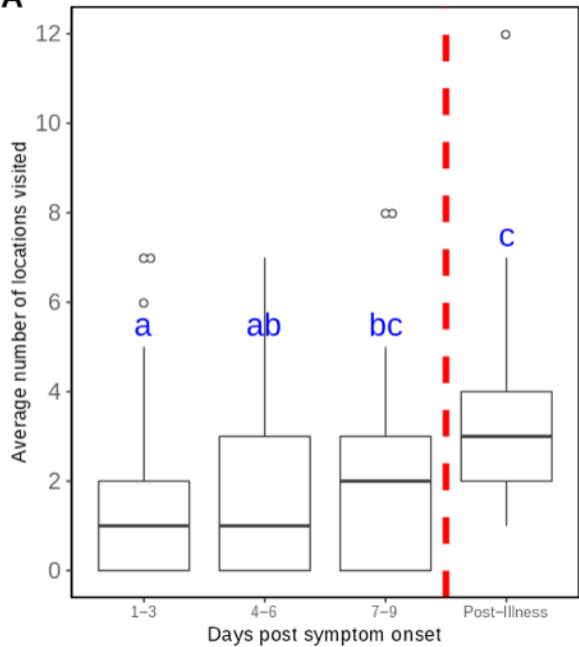**B**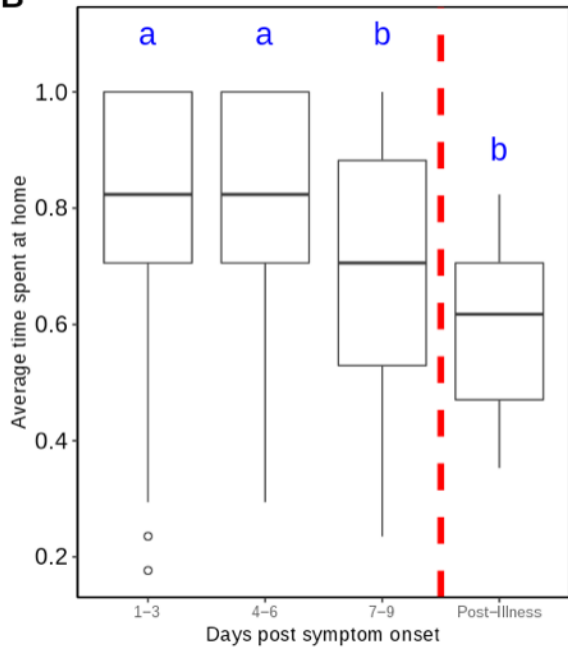

Supplement: S2 Fig — (A) Average number of locations visited per 3-day period. (B) Average proportion of time spent at home per 3-day period. Significant differences, denoted by letters, were detected using pairwise paired Wilcoxon Sign Rank tests with Bonferroni’s correction to account for a family-wise error-rate of 0.05. All significant differences had p-values < 0.05. (PDF) [file pcbi.1008627.s024.pdf]

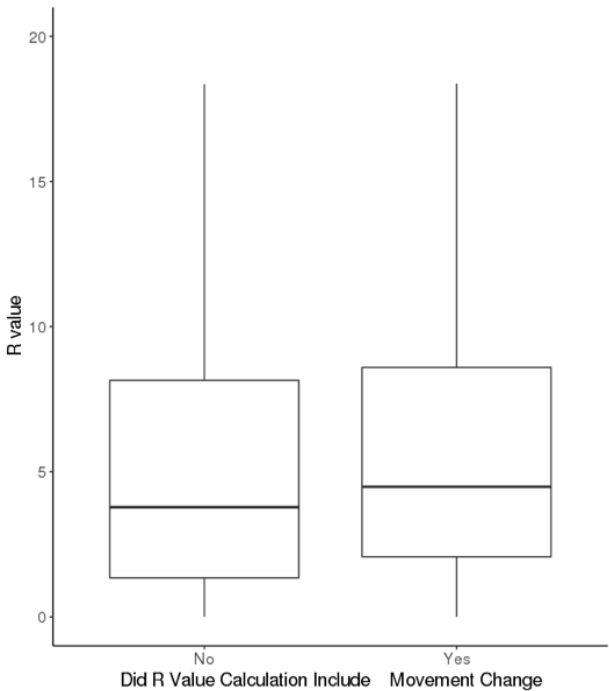

Supplement: S3 Fig — Outliers were removed. (PDF) [file pcbi.1008627.s025.pdf]

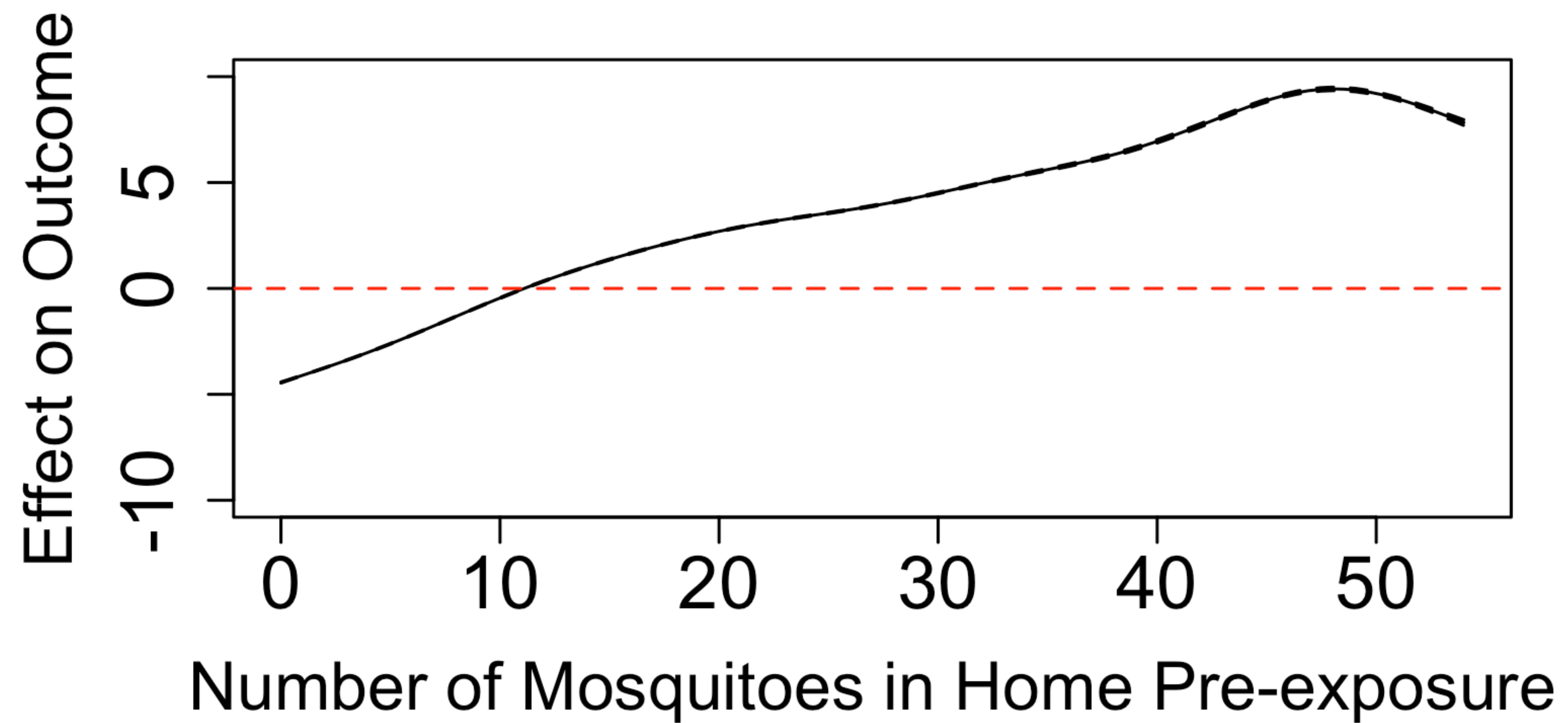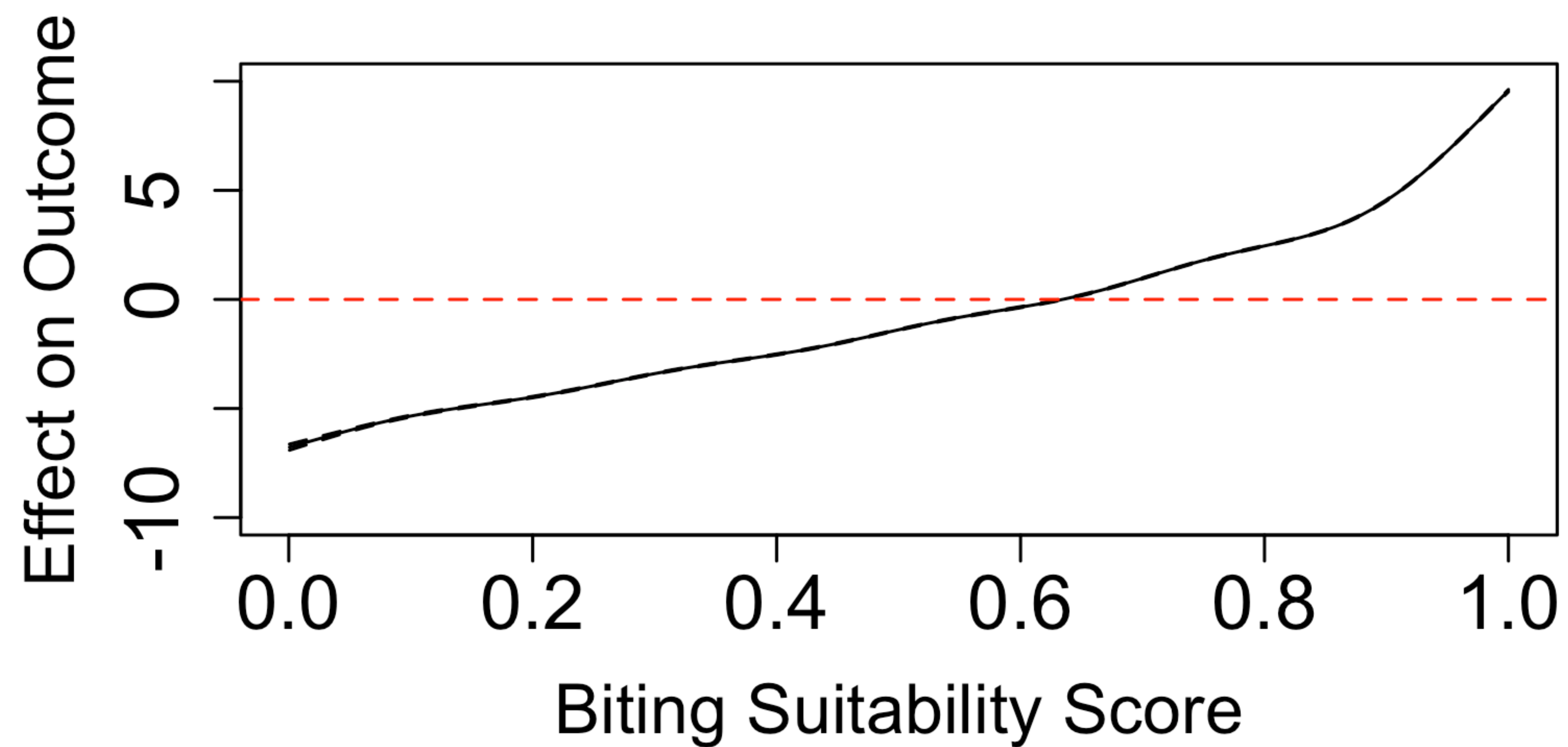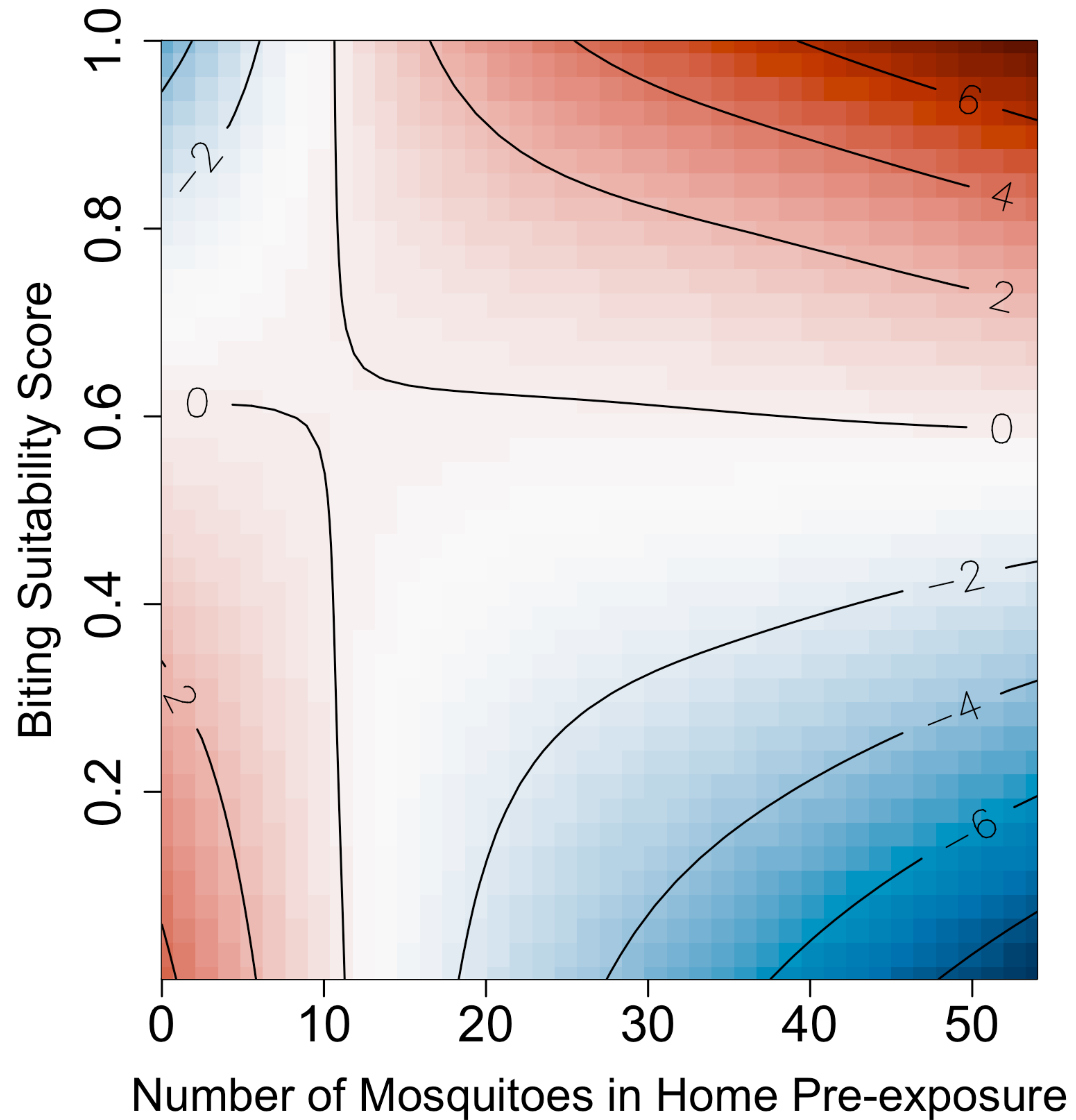

Supplement: S4 Fig — Smooth functions for Rmovement based on a GAM model containing number of mosquitoes in home pre-exposure, biting suitability score, and their interaction. The component smooths for each predictor variable are provided. For the 1-d smooths, the y-axis is the contribution of the predictor variable to the fitted response, centered around 0 (with 0 denoted by a red dashed line). For the 2-d smooth for the interaction term, a heatmap with overlaid contours is provided. The values of the contours represent the contribution of the interaction term to the fitted response. Positive values are in red and negative values are in blue. (PDF) [file pcbi.1008627.s026.pdf]

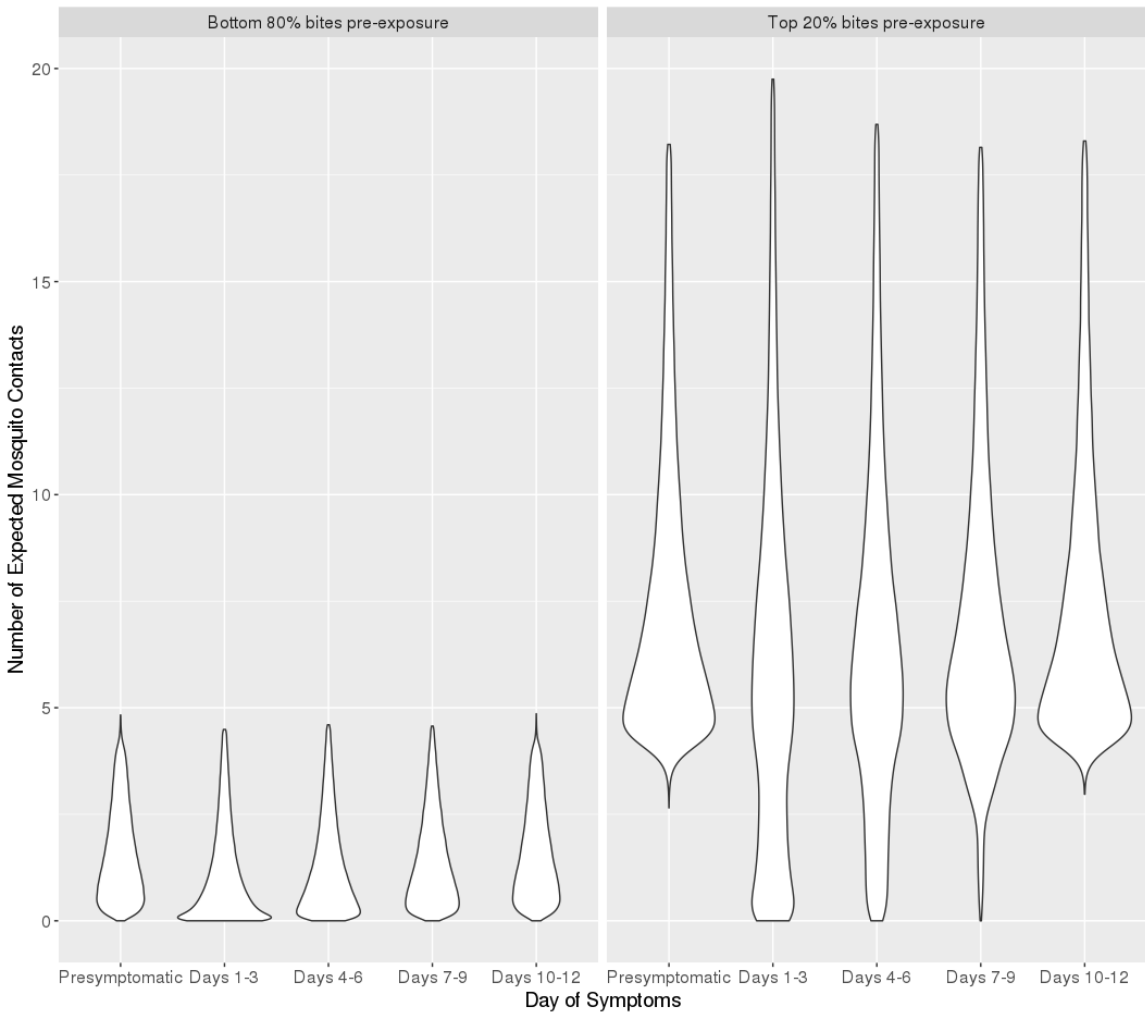

Supplement: S5 Fig — (PDF) [file pcbi.1008627.s027.pdf]

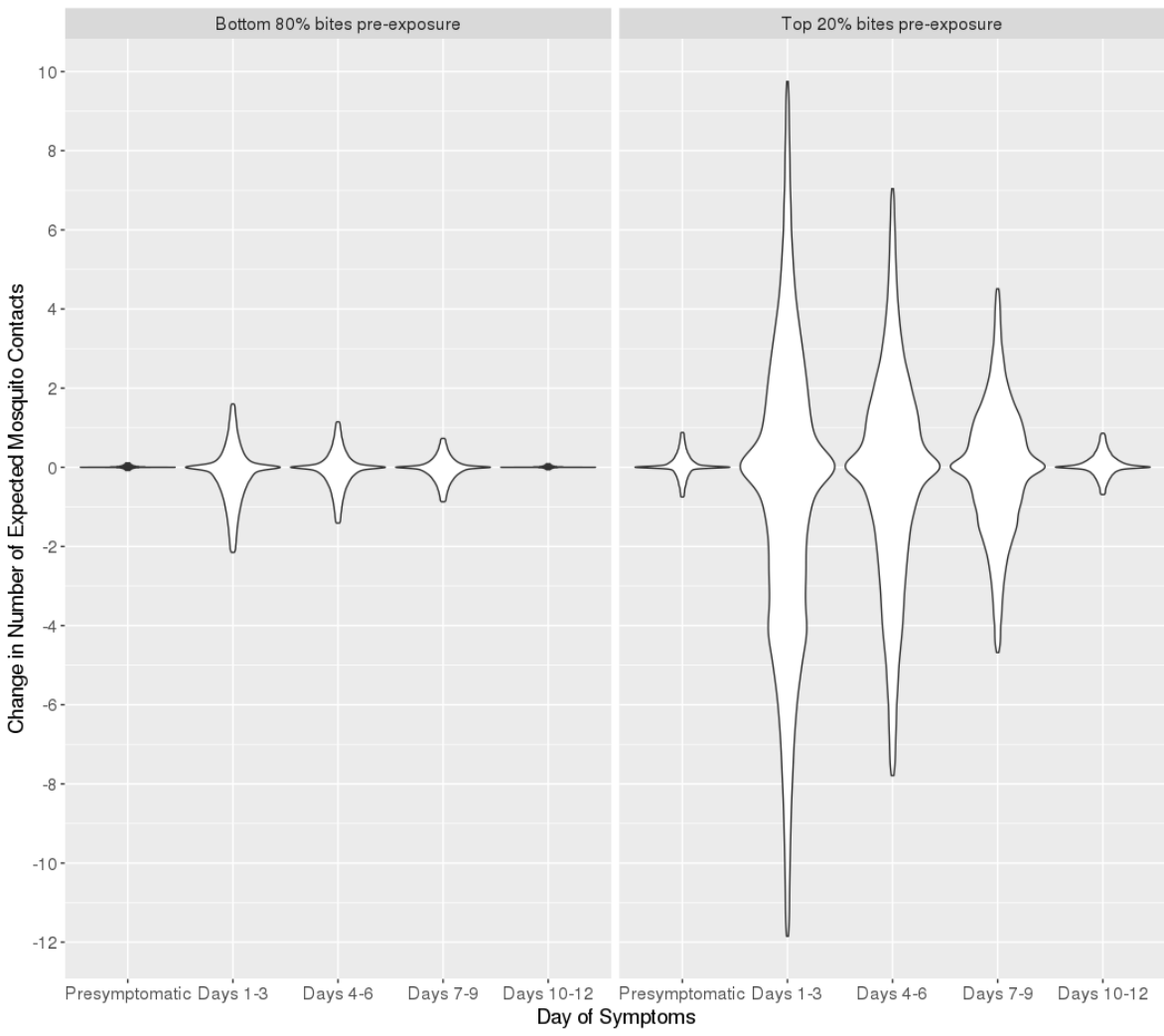

Supplement: S6 Fig — (PDF) [file pcbi.1008627.s028.pdf]

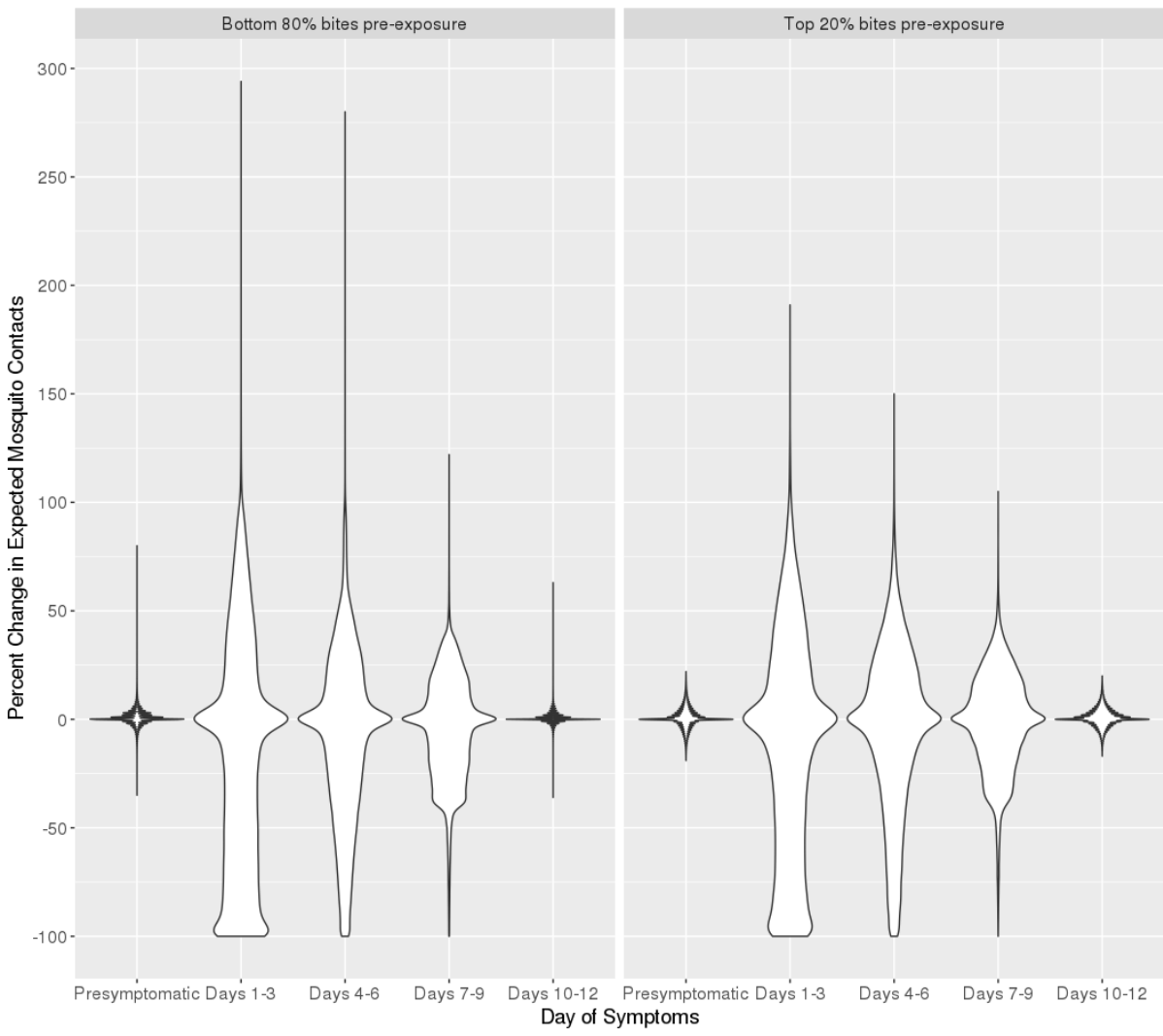

Supplement: S7 Fig — (PDF) [file pcbi.1008627.s029.pdf]

Expected Percent Change in Mosquito Contacts

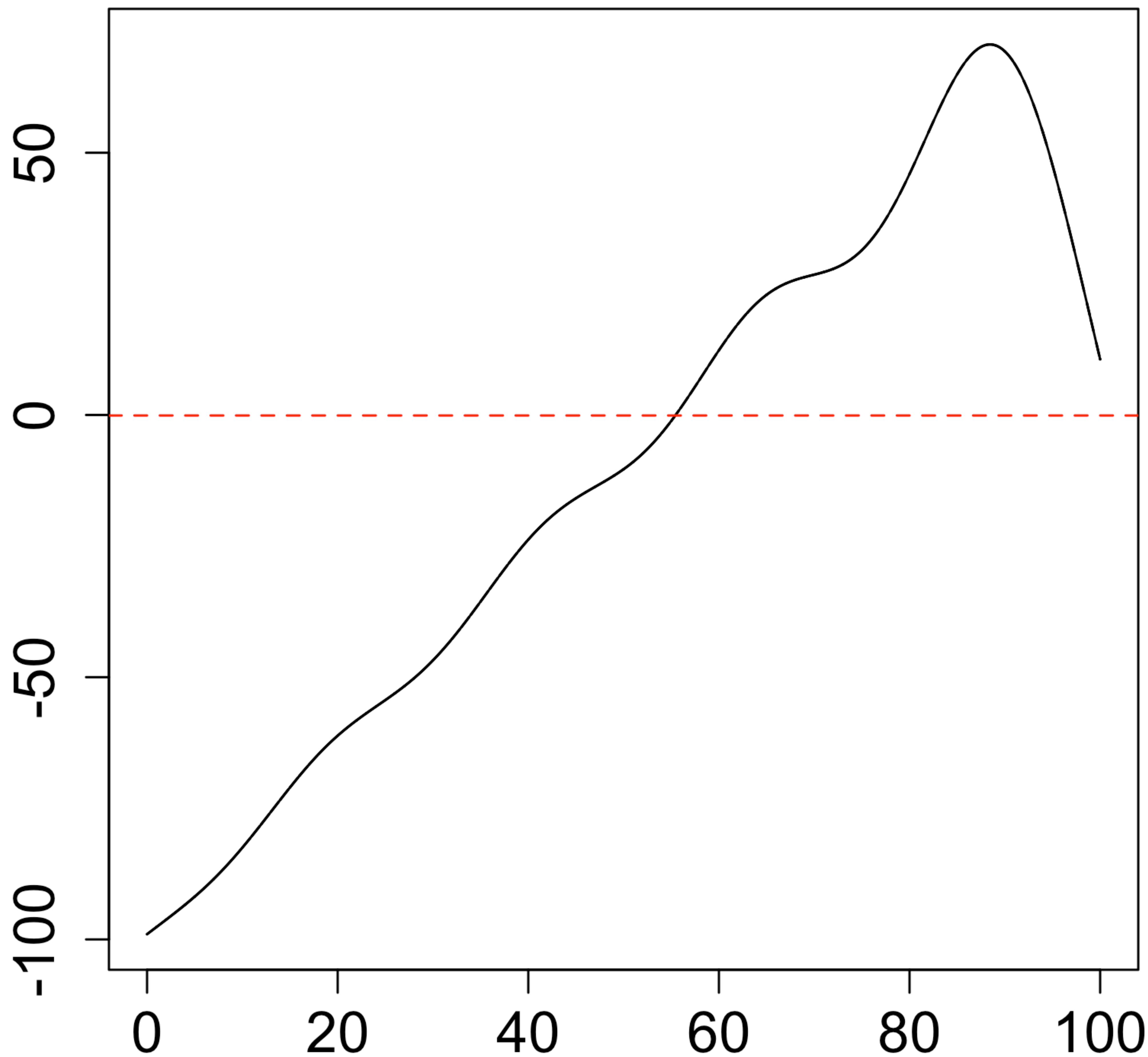

Percent of Bites Expected at Home Pre-exposure

Supplement: S8 Fig — The predicted percent change in expected bites based on percent of bites expected at home pre-exposure. As there is only one predictor variable in this model, shifting the y-axis by the intercept value provides the smooth for the predictor variable. (PDF) [file pcbi.1008627.s030.pdf]

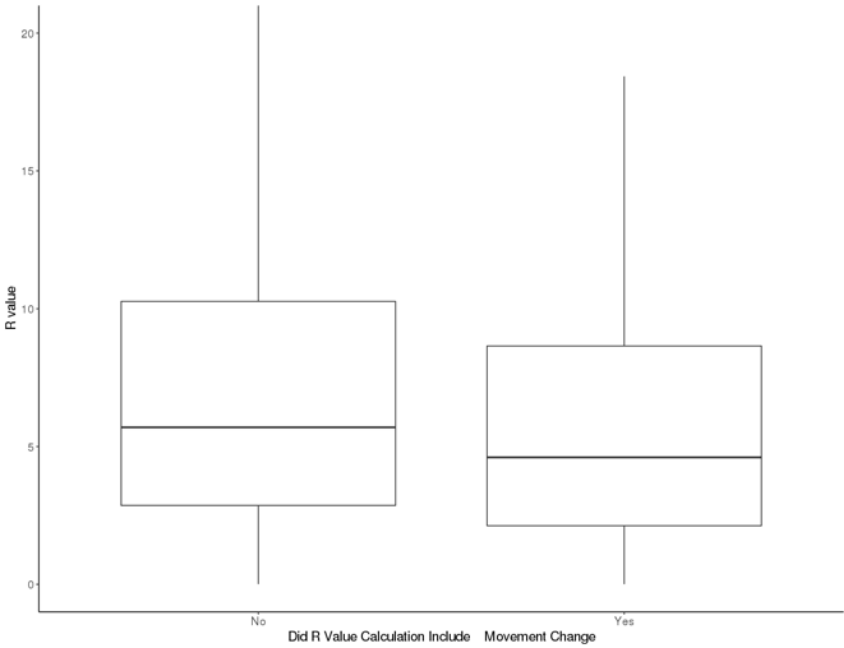

Supplement: S9 Fig — Outliers were removed. (PDF) [file pcbi.1008627.s031.pdf]

**A**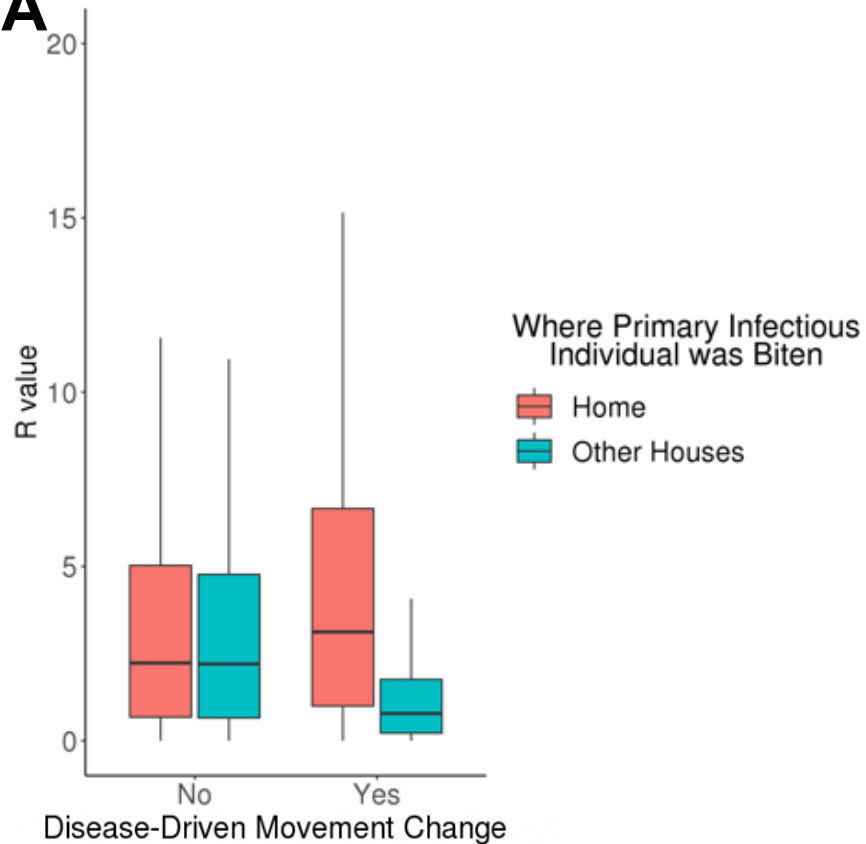**B**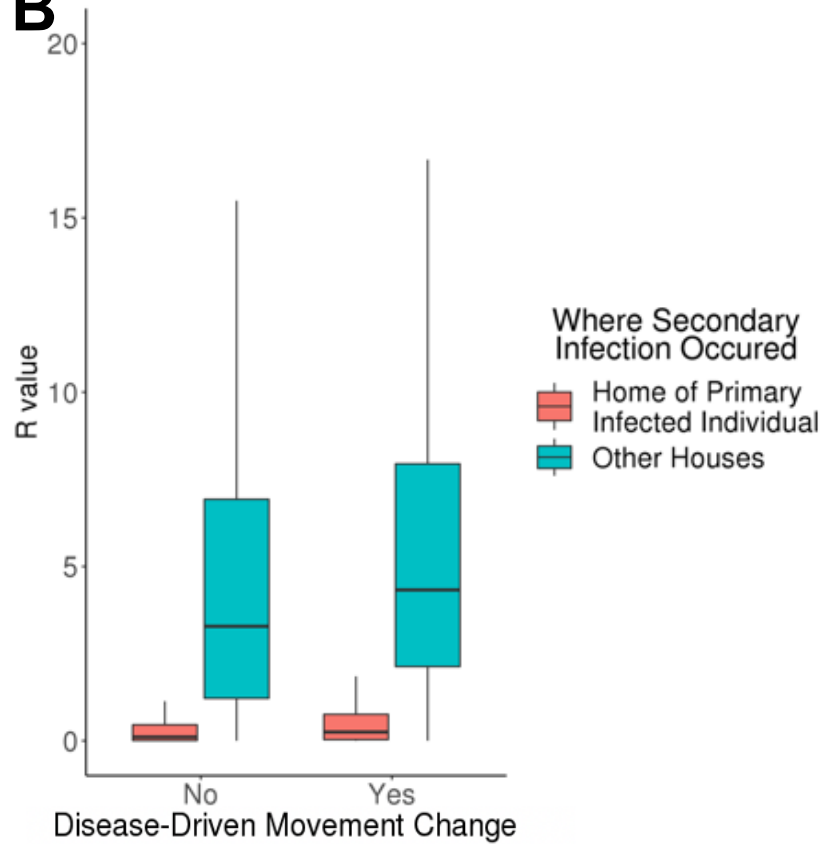

Supplement: S10 Fig — (A) gives onward transmission for primary bites occurring at home (red) and at other houses (blue) both without (left) and with (right) movement change included. [from left to right: Rnorm(home), Rnorm(other houses), Rmovement(home), and Rmovement(other houses)] (B) gives onward transmission for secondary bites at the home of the primary infected individual (red) and at other houses (blue). (PDF) [file pcbi.1008627.s032.pdf]

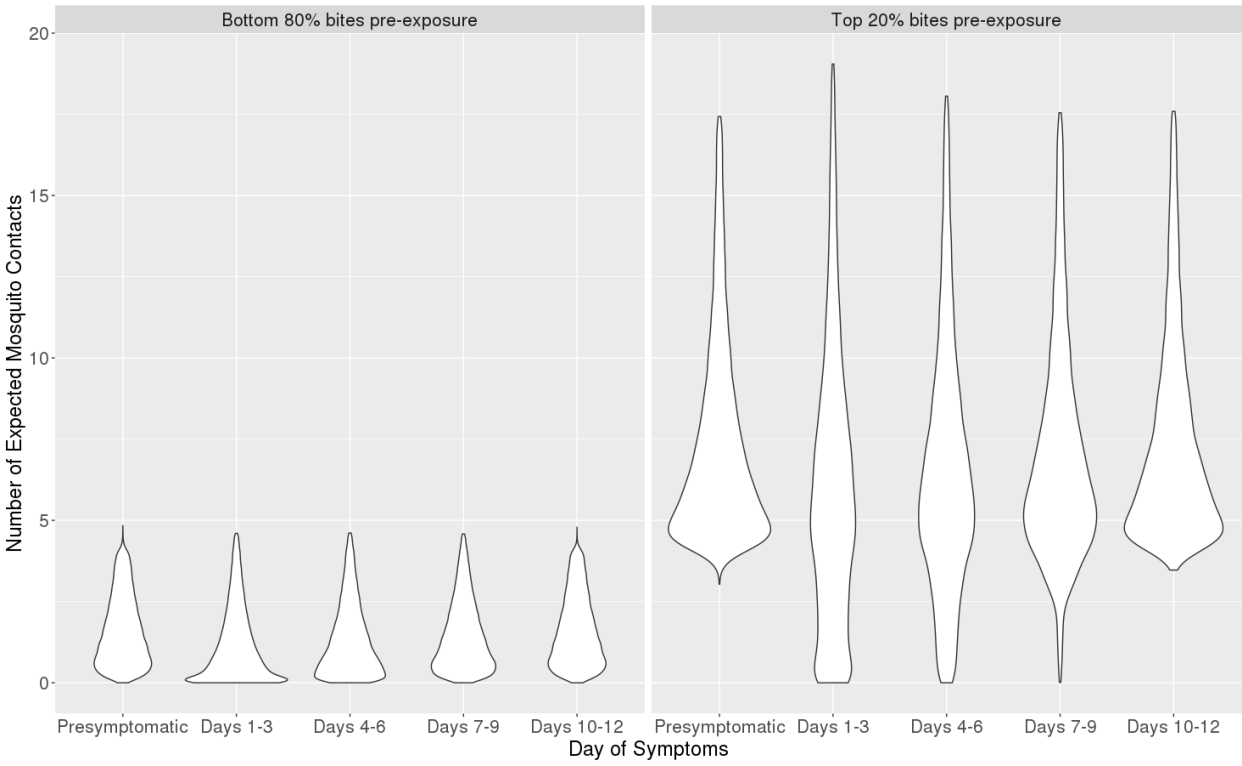

Supplement: S11 Fig — (PDF) [file pcbi.1008627.s033.pdf]

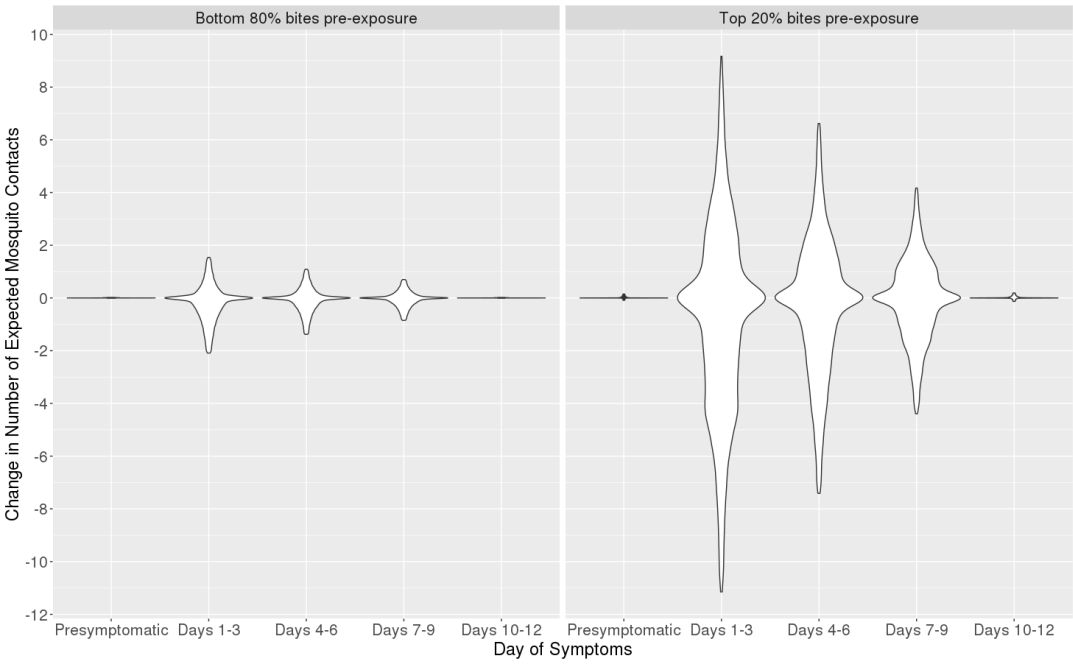

Supplement: S12 Fig — (PDF) [file pcbi.1008627.s034.pdf]

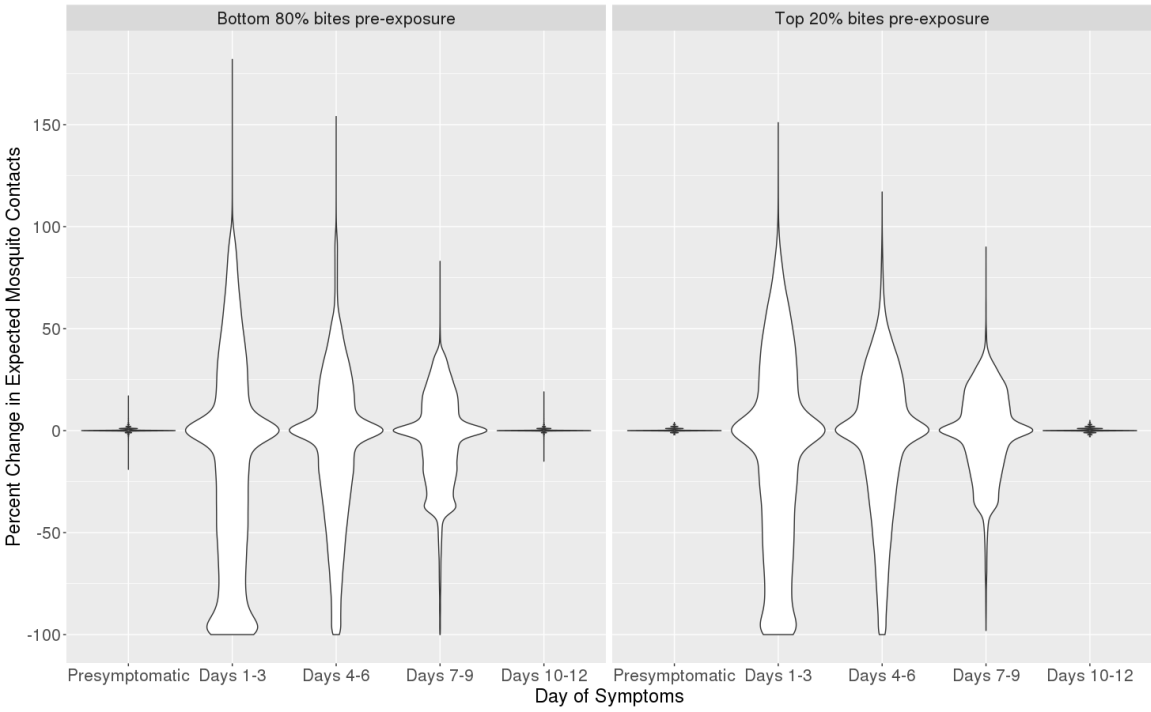

Supplement: S13 Fig — (PDF) [file pcbi.1008627.s035.pdf]

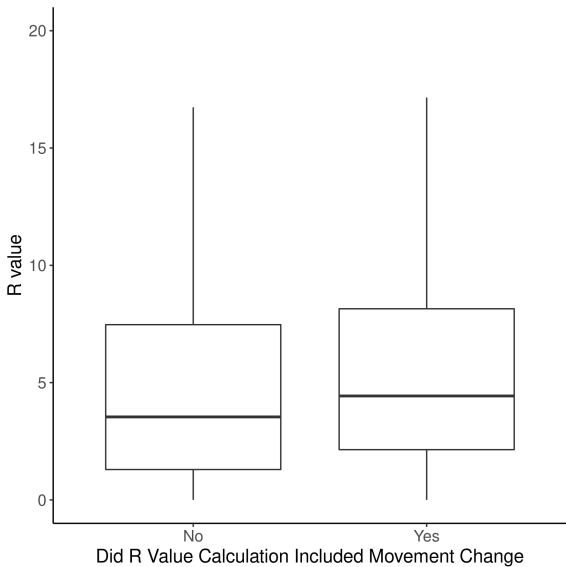

Supplement: S14 Fig — Outliers were removed. (PDF) [file pcbi.1008627.s036.pdf]

**A**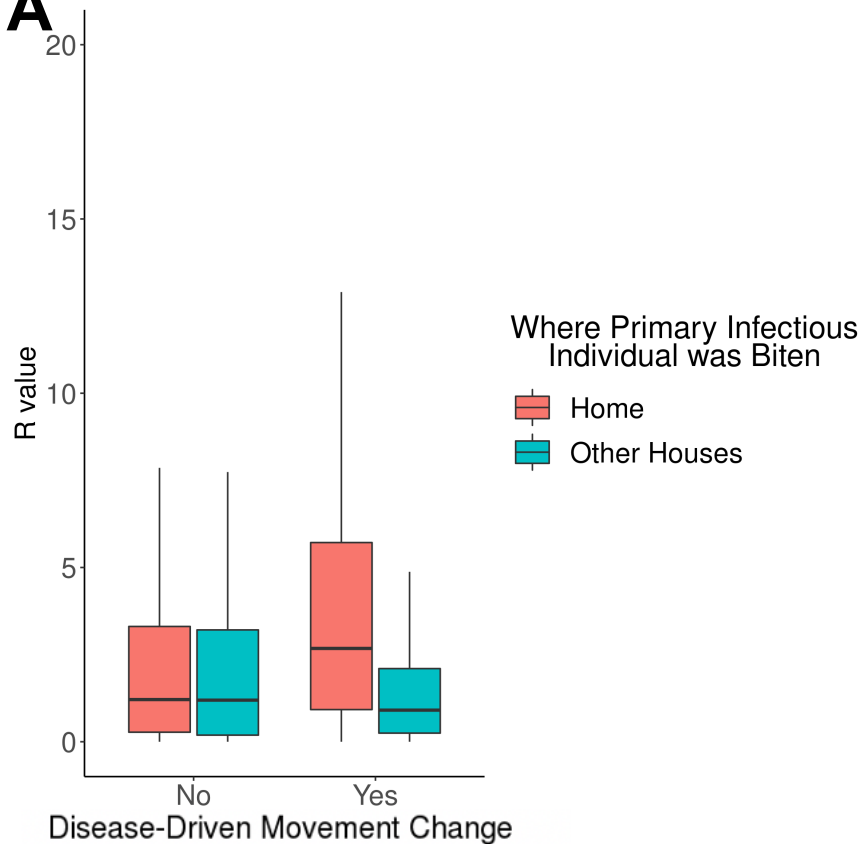**B**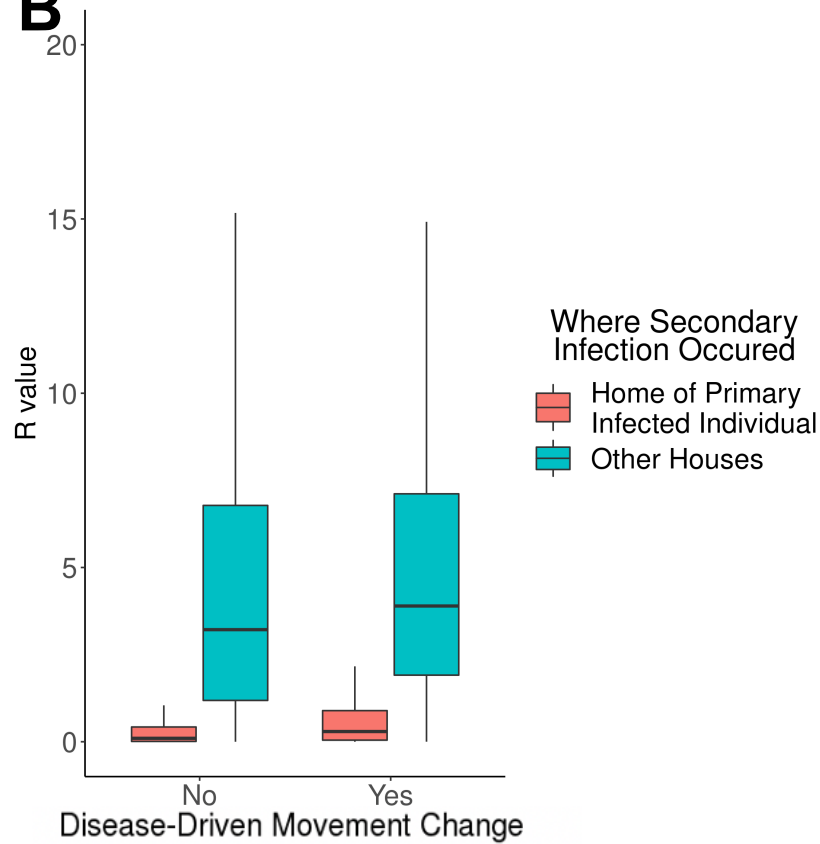

Supplement: S15 Fig — (A) gives onward transmission for primary bites occurring at home (red) and at other houses (blue) both without (left) and with (right) movement change included. [from left to right: Rnorm(home), Rnorm(other houses), Rmovement(home), and Rmovement(other houses)] (B) gives onward transmission for secondary bites at the home of the primary infected individual (red) and at other houses (blue). (PDF) [file pcbi.1008627.s037.pdf]
