# Supplementary material for: Disease-driven reduction in human mobility influences human-mosquito contacts and dengue transmission dynamics
Source: PLoS Comput Biol. 2021 Jan 19;17(1):e1008627. doi: 10.1371/journal.pcbi.1008627 (PMC7845972; doi:10.1371/journal.pcbi.1008627)
Supplement: S18 Fig — (PDF) [file pcbi.1008627.s040.pdf]

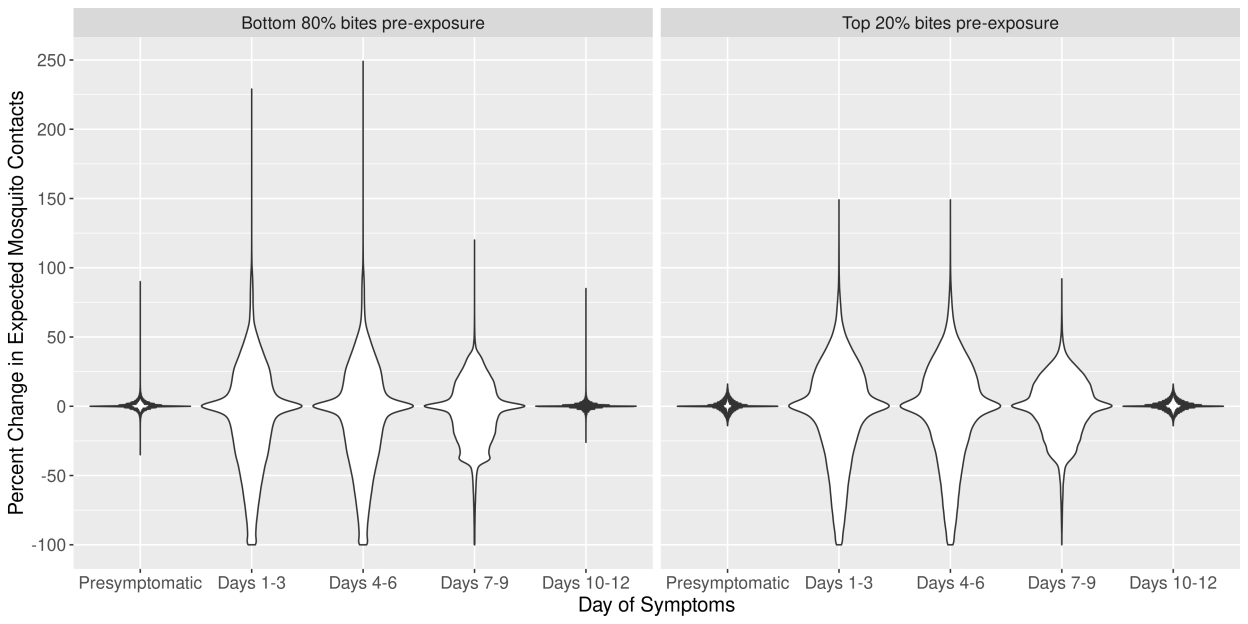
Bottom 80% bites pre-exposure
Top 20% bites pre-exposure
Percent Change in Expected Mosquito Contacts
250
200
150
100
50
0
-50
-100
Presymptomatic
Days 1-3
Days 4-6
Days 7-9
Days 10-12
Day of Symptoms
